# A multicenter study of artificial intelligence-aided software for detecting visible clinically significant prostate cancer on mpMRI

**DOI:** 10.1186/s13244-023-01421-w

**Published:** 2023-04-30

**Authors:** Zhaonan Sun, Kexin Wang, Zixuan Kong, Zhangli Xing, Yuntian Chen, Ning Luo, Yang Yu, Bin Song, Pengsheng Wu, Xiangpeng Wang, Xiaodong Zhang, Xiaoying Wang

**Affiliations:** 1 Department of Radiology, Peking University First Hospital, No.8 Xishiku Street, Xicheng District, Beijing, 100034 China; 2 School of Basic Medical Sciences, Capital Medical University, Beijing, China; 3 Department of Radiology, The Second Affiliated Hospital of Dalian Medical University, Dalian, Liaoning, China; 4 Department of Radiology, Fujian Medical University Union Hospital, Fuzhou, Fujian China; 5 Department of Radiology, West China Hospital, Sichuan University, Chengdu, Sichuan China; 6 Beijing Smart Tree Medical Technology Co. Ltd., Beijing, China

**Keywords:** Prostatic neoplasms, Deep learning, Diagnosis, Computer-assisted, Magnetic resonance imaging

## Abstract

**Background:**

AI-based software may improve the performance of radiologists when detecting clinically significant prostate cancer (csPCa). This study aims to compare the performance of radiologists in detecting MRI-visible csPCa on MRI with and without AI-based software.

**Materials and methods:**

In total, 480 multiparametric MRI (mpMRI) images were retrospectively collected from eleven different MR devices, with 349 csPCa lesions in 180 (37.5%) cases. The csPCa areas were annotated based on pathology. Sixteen radiologists from four hospitals participated in reading. Each radiologist was randomly assigned to 30 cases and diagnosed twice. Half cases were interpreted without AI, and the other half were interpreted with AI. After four weeks, the cases were read again in switched mode. The mean diagnostic performance was compared using sensitivity and specificity on lesion level and patient level. The median reading time and diagnostic confidence were assessed.

**Results:**

On lesion level, AI-aided improved the sensitivity from 40.1% to 59.0% (18.9% increased; 95% confidence interval (CI) [11.5, 26.1]; *p* < .001). On patient level, AI-aided improved the specificity from 57.7 to 71.7% (14.0% increase, 95% CI [6.4, 21.4]; *p* < .001) while preserving the sensitivity (88.3% vs. 93.9%, *p* = 0.06). AI-aided reduced the median reading time of one case by 56.3% from 423 to 185 s (238-s decrease, 95% CI [219, 260]; *p* < .001), and the median diagnostic confidence score was increased by 10.3% from 3.9 to 4.3 (0.4-score increase, 95% CI [0.3, 0.5]; *p* < .001).

**Conclusions:**

AI software improves the performance of radiologists by reducing false positive detection of prostate cancer patients and also improving reading times and diagnostic confidence.

**Clinical relevance statement:**

This study involves the process of data collection, randomization and crossover reading procedure.

**Graphical Abstract:**

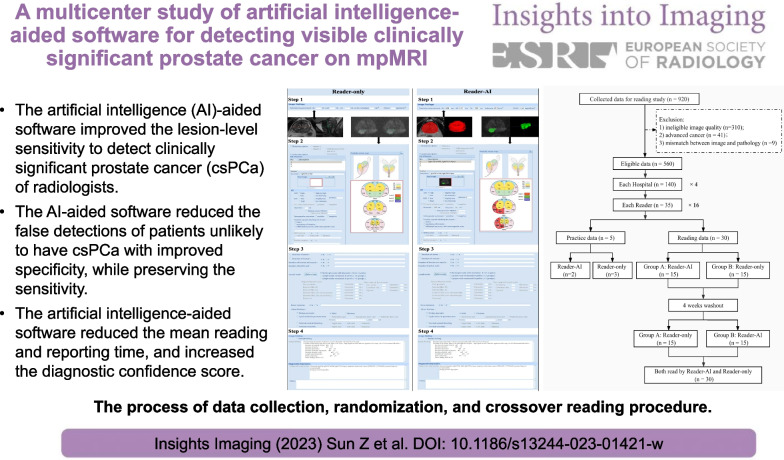

**Supplementary Information:**

The online version contains supplementary material available at 10.1186/s13244-023-01421-w.

## Key points

- The artificial intelligence (AI)-aided software improved the lesion-level sensitivity to detect MRI- visible clinically significant prostate cancer (csPCa) of radiologists.
- The AI-aided software reduced the false positive detection of prostate cancer patients with improved specificity while preserving the sensitivity.
- The AI-aided software reduced the mean reading and reporting time and increased the diagnostic confidence score for MRI-visible csPCa diagnosis.

**Supplementary Information:**

The online version contains supplementary material available at 10.1186/s13244-023-01421-w.

## Introduction

Multiparametric MRI (mpMRI), as a noninvasive triage tool, can not only detect clinically significant prostate cancer (csPCa) lesions but also provide information on locoregional staging and biopsy [1–3]. Combined with the test of serum prostate-specific antigen (PSA), an “MRI diagnosis pathway” [4] may have the potential to mitigate excessive biopsy [5] and consequent overtreatment for indolent lesions [6]. Thus, the European Association of Urology has recommended mpMRI as the backbone for the primary prostate cancer diagnostic pipeline to properly identify candidates for image-guided biopsy [7].

The Prostate Imaging Reporting and Data System (PI-RADS) [8] has been launched to guide standardized acquisition, interpretation and reporting procedures for prostate mpMRI [9]. As shown in the meta-analysis [10], a pooled sensitivity of 0.89 and a specificity of 0.73 were shown for PI-RADS Version 2 in detecting visible csPCa. Since approximately half of the tumor foci are MRI-invisible, the sensitivity of detection at the lesion level is much lower [11, 12]. Even with visible lesions, PI-RADS performance is not optimal due to the high inter-rater and intra-rater variability, and a high degree of expertise is required [13–16].

Recently, many computer-aided detection (CAD) systems on mpMRI have shown good performance in prostate cancer diagnosis [17]. CAD systems can enhance radiologists diagnostic performance and reduce interpretation inconsistencies. Many studies [18–21] suggested that AI-based CAD systems have potential clinical utility in csPCa detection. However, the performances of CAD systems reported in these studies may be dataset-specific, and their generalization, that is, performance on outside datasets, has not been well studied. To this end, it is necessary for external validation in a multicenter, multivendor clinical setting before the CAD systems are applied to radiologists’ workflow.

In this study, previously trained AI algorithms were embedded into a proprietary structured reporting software, and radiologists simulated their real-life work scenarios to interpret and report the PI-RADS category of each case using this AI-based software. The purpose of this study is to compare the diagnostic performance, reading and reporting time, and diagnostic confidence of radiologists in detecting MRI-visible csPCa on MRI with and without AI-based software.

## Materials and methods

Radiologists from four hospitals participated in this study. The mpMRI images were retrospectively gathered from three hospitals. No study data were used in the previous development of the AI models.

### Study dataset

We collected mpMRI images from three hospitals (Peking University First Hospital, the Second Affiliated Hospital of Dalian Medical University and Fujian Medical University Union Hospital) between June 2017 and August 2018. All patients had clinical indications for prostate mpMRI examination, underwent both TRUS-guided systematic (12- or 6-core needles) and targeted biopsy after mpMRI examination, and no prostate cancer-related treatment was performed before the examination. Eleven different MR scanners were used in the four hospitals for the acquisition of prostate mpMRI. The detailed protocols of mpMRI are shown in Table 1. The correlated clinical information was also collected, including PSA value, pathological results and clinical follow-up results. Exclusion criteria were (a) ineligible image quality, (b) cases showing obvious extracapsular extension, diffuse pelvic lymph adenopathy and/or bone metastasis, and (c) mismatch between the mpMRI image and the pathology result, which includes cancer that is not visible on imaging and images that show cancer but are pathologically negative.

**Table 1:** MR imaging protocol

	Hospital_1 (N = 332)	Hospital_3 (N = 63)	Hospital_4 (N = 85)	Overall (N = 480)
*Manufacture, n (%)*				
GE	177 (53.3)	43 (68.3)	51 (60.0)	271 (56.5)
Philips	45 (13.6)	10 (15.9)	6 (7.1)	61 (12.7)
SIEMENS	107 (32.2)	7 (11.1)	28 (32.9)	142 (29.6)
UIH	3(0.9)	3 (4.8)	0 (0)	6 (1.3)
*Magnetic field, n (%)*				
1.5 T	15 (4.5)	7 (11.1)	3 (3.5)	25 (5.2)
3.0 T	317 (95.5)	56 (88.9)	82 (96.5)	455 (94.8)
*Model name, n (%)*				
Achieva	8 (2.4)	2 (3.2)	3 (3.5)	13 (2.7)
Aera	15 (4.5)	7 (11.1)	0 (0)	22 (4.6)
DISCOVERY MR750	158 (47.6)	43 (68.3)	42 (49.4)	243 (50.6)
DISCOVERY MR750w	19 (5.7)	0 (0)	9 (10.6)	28 (5.8)
Ingenia	37 (11.1)	8 (12.7)	0 (0)	45 (9.4)
Multiva	0 (0)	0 (0)	3 (3.5)	3 (0.6)
Prisma	10 (3.0)	0 (0)	3 (3.5)	13 (2.7)
Skyra	14 (4.2)	0 (0)	5 (5.9)	19 (4.0)
TrioTim	49 (14.8)	0 (0)	19 (22.4)	68 (14.2)
uMR 790	3 (0.9)	3 (4.8)	0 (0)	6 (1.3)
Verio	19 (5.7)	0 (0)	1 (1.2)	20 (4.2)
*Scanning sequence, n (%)*				
EP	76 (22.9)	3 (4.8)	27 (31.8)	106 (22.1)
EP/SE	177 (53.3)	43 (68.3)	51 (60.0)	271 (56.5)
RM	15 (4.5)	7 (11.1)	0 (0)	22 (4.6)
SE	45 (13.6)	10 (15.9)	6 (7.1)	61 (12.7)
Missing	19 (5.7)	0 (0)	1 (1.2)	20 (4.2)
*B value, n (%)*				
800	3 (0.9)	2 (3.2)	1 (1.2)	6 (1.3)
1000	0 (0)	28 (44.4)	3 (3.5)	31 (6.5)
1200	0 (0)	14 (22.2)	2 (2.4)	16 (3.3)
1400	329 (99.1)	6 (9.5)	0 (0)	335 (69.8)
1500	0 (0)	13 (20.6)	67 (78.8)	80 (16.7)
2000	0 (0)	0 (0)	12 (14.1)	12 (2.5)
Slice thickness, mm	4.0 [3.0, 5.0]	4.0 [3.0, 5.0]	4.0 [3.0, 5.0]	4.0 [3.0, 5.0]
Slice spacing, mm	4.0 [3.0, 6.5]	4.0 [3.3, 5.0]	4.0 [3.0, 5.5]	4.0 [3.0, 6.5]
Pixel spacing, mm	0.9 [0.8, 4.2]	0.9 [0.8, 2.1]	0.9 [0.6, 3.6]	0.9 [0.6, 4.2]
Matrix size—rows	256 [59, 283]	256 [96, 256]	256 [53, 256]	256 [53, 283]
Matrix size—columns	256 [96, 256]	256 [96, 256]	256 [136, 256]	256 [96, 256]
FOV 1, mm	225 [55, 533]	240 [90, 533]	240 [52, 533]	240 [52, 533]
FOV2, mm	225 [75, 533]	240 [90, 533]	240 [134, 533]	240 [75, 533]
TR, ms	2669 [2000, 6759]	4077 [2200, 6100]	2500 [2500, 5300]	2671 [2000, 6759]
TE, ms	61 [51, 90]	73 [62, 93]	91 [57, 91]	61 [51, 93]

EP = echo planar imaging, SE = spin-echo imaging, RM = ramp sampling, FOV = field of view, TR = repetition time, TE = echo time. The categorical variables are given as absolute frequency (relative frequency). Quantitative variables were given as the median [minimum, maximum] for nonnormalized data

### Reference standard

A combined pathology [22] was created from the systematic and targeted biopsy and used as reference standard. A total of 12 dedicated urologists (10–35-year experience in prostate biopsies) from three hospitals performed the prostate biopsy using the following same biopsy techniques with their own hardware, i.e., double-plane B-ultrasounds (LOGIQ E9, GE; EPIQ 7, Philips; Hivision Ascendus, Hitachi; RS80A, Samsung), transrectal probes and corresponding puncture needle guns. For system biopsy, 12- or 6-core needles biopsies were adopted. For the targeted biopsy, based on structured reports prepared by dedicated urogenital radiologists during the clinical routine, lesions suspected of malignancy were marked on a prostate sector map [23] for targeted biopsy. At least one urologist and one urogenital radiologist would review MR images before biopsy in a multidisciplinary meeting to ensure accurate localization of suspicious lesions. When performing biopsies, the urologists examined each suspicious lesion with an additional needle core (2 to 5-core needles). A total of 9 dedicated genitourinary pathologists (8- to 30-year experience in prostate pathology interpretation) analyzed and recorded the histopathology on each specimen. The criteria for a negative case were negative biopsy and no prostate cancer at more than one year of clinical follow-up. The criteria for a positive case were positive pathology with a Gleason score ≥ 7 or a Gleason score of 3 + 3 with volume ≥ 0.5 cc. For positive cases, the two uro-radiologists (Z.S. and X.W. with 4 and 30 years of experience in prostate MRI diagnosis) mapped the pathological ground truth of each csPCa focus to the diffusion-weighted imaging and annotated them with consensus. The open-source software ITK-SNAP [24] (version 3.8.0 2019; available at www.itksnap.org) was used to annotate the tumor foci.

### AI software description

A proprietary deep learning-based AI software was used for the study. It consists of four AI models: (i) MRI sequence classification, (ii) prostate gland segmentation and measurement [25], (iii) prostate zonal anatomy segmentation and (iv) csPCa foci segmentation and measurement. Additional file 1 shows detailed information on the development and performance of the AI software. The models were sequentially executed, and the results were automatically input into the PI-RADS structured report [26].

### Reporting with the structured reporting software

**Fig. 1:**
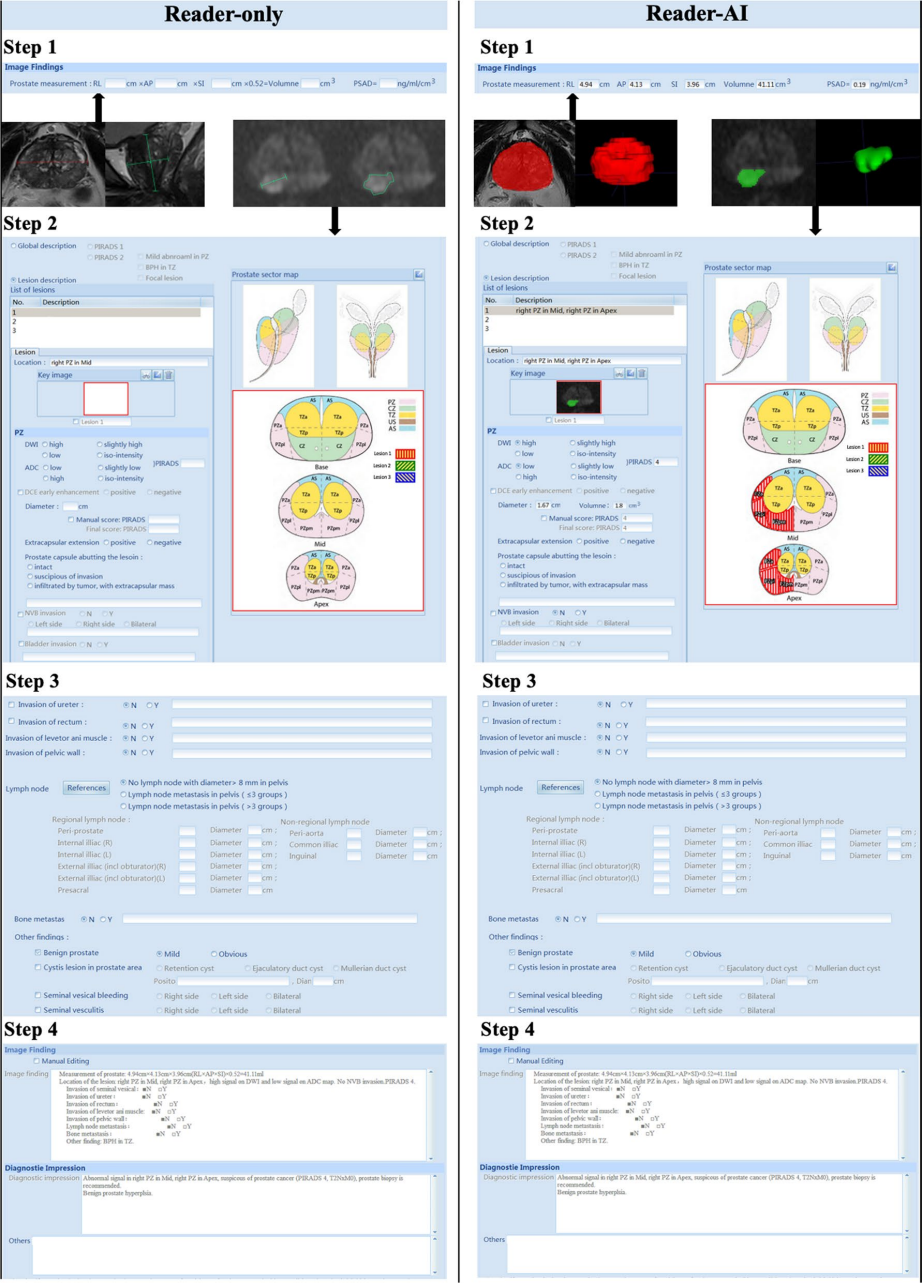
Process of Reader-only and Reader-AI using structured reporting software

Sixteen radiologists (1–5-year experience in prostate mpMRI interpretation) from four hospitals (Peking University First Hospital, the Second Affiliated Hospital of Dalian Medical University, Fujian Medical University Union Hospital and Sichuan University West China Hospital) were invited as readers. They were familiar with the PI-RADS (version 2.1) guideline and followed it in their practical work. Six of them had experience with more than 100 cases, two had experience with 50–100 cases, and the other eight radiologists had experience with less than 50 cases. The readers were blinded to all patients’ clinical information. We used structured reporting software to read and record the results. Before the study, they were trained to use the reporting software with 80 practice cases outside the study data. Each reader had five practice cases, of which two were read with AI assistance, and three were read without AI assistance. Figure 1 illustrates the process of Reader-only and Reader-AI using the structured reporting software.

The Reader-only mode included four steps. The first step measures the diameters of the prostate gland. The readers measured the transverse, anterior–posterior and cranio–caudal lengths of the prostate gland. The second step is to detect and measure the suspected lesion. The readers recorded up to four largest PI-RADS ≥ 3 lesions. They recorded the location, measured the maximum diameter and gave a PI-RADS score to each lesion. The third step evaluates other findings. The readers recorded other findings as they did in their clinical practice, including invasion of surrounding structures and other benign findings. The fourth step gives the overall impression. The readers summarized all the findings and gave a global expression. The readers rated their diagnostic confidence for each case on a 5-point scale (1 ≤ 25%, 2 = 25–50%, 3 = 50–75%, 4 = 75–90%, 5 ≥ 90%) [27]. The reading and reporting time of each case was automatically recorded by the software.

The Reader-AI mode followed the same process; the only difference was that there was AI help in the first and second steps. When the readers opened the patient list, the prostate gland and the suspicious lesions were already annotated and highlighted by the AI software. The readers might approve, reject or amend the AI findings at their discretion.

Lesions with PI-RADS scores higher than or equal to 3 were considered positive for csPCa lesions. Patients with at least one positive csPCa lesion were considered positive, and patients with no csPCa lesion were considered negative.

### Crossover reading method

The 480 cases were randomly divided into two groups: group A and group B. We assigned the two groups of cases to the 16 readers, i.e., each reader received 30 cases, with 15 in group A and 15 cases in group B.

**Fig. 2:**
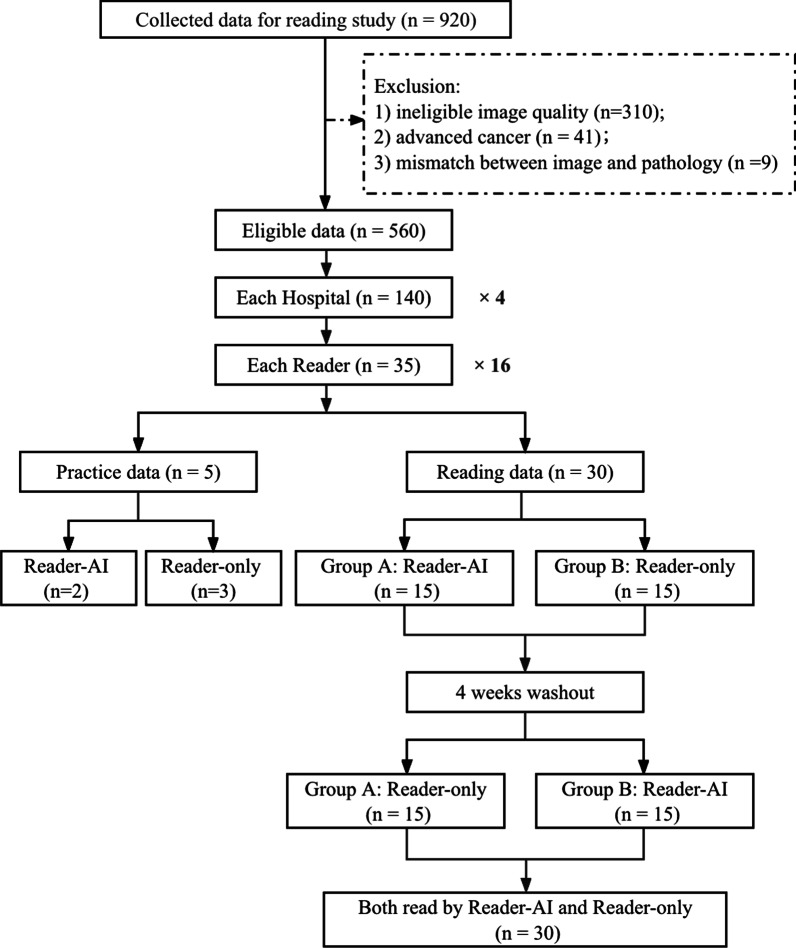
Flow diagram of data collection, randomization and crossover reading procedure. The data were randomly divided into two parts, Group A and Group B. Advanced cancer means cases with obvious extracapsular extension, diffuse pelvic lymph adenopathy and/or bone metastasis

The reading study was conducted in two reading sessions with an interval of four weeks. In the first reading session, cases in group A were read with Reader-AI mode, while cases in group B were read with Reader-only mode. In the second reading session, cases in group A were read with Reader-only mode, while cases in Group B were read with Reader-AI mode. The crossover reading procedure is shown in Fig. 2.

### Statistical analysis

All statistical tests were performed using R 4.2.0 (Comprehensive R Archive Network, www.r-project.org). Quantitative variables were given as the mean (standard deviation) for normalized data and as the median [minimum, maximum] for nonnormalized data. The categorical variables are given as absolute frequency (relative frequency). The mean lesion-level sensitivity, patient-level sensitivity and specificity with 95% confidence intervals (CIs) across all 16 radiologists of the two reading modes were computed and then compared by the Chi-square test. The median reading and reporting time, and diagnostic confidence were compared by the Wilcoxon rank-sum test. All statistical tests were two-tailed with a 5% level of statistical significance.

## Results

### Clinical characteristics

A total of 480 cases were included in this study with a serum total PSA value of 7.69 [0.150, 100] ng/ml. A total of 180 (37.5%) cases were proved to be csPCa, with 349 MRI-visible csPCa lesions. Of the 180 positive cases, 39 cases had lesions located only in the peripheral zone, 28 cases had lesions located only in the transition zone, and 113 cases had lesions located in both peripheral and transition zones. The lesion volumes were calculated by summing the pixel volumes within the annotated areas of the reference standard. The median volume of MRI-visible csPCa lesions was 2.0 [1.0, 4.8] cm^3^. The clinical and demographic characteristics of the eligible cases are shown in Table 2.

**Table 2:** Clinical and demographic characteristics of eligible cases

	Hospital_1 (N = 332)	Hospital_2 (N = 63)	Hospital_3 (N = 85)	Overall (N = 480)
*Reference standards, n (%)*				
Negative	263 (79.2)	15 (23.8)	22 (25.9)	300 (62.5)
Positive	69 (20.8)	48 (76.2)	63 (74.1)	180 (37.5)
Age (years)	65.5 ± 11.0	70.4 ± 8.1	69.3 ± 7.4	66.8 ± 10.2
tPSA (ng/ml)	6.5 [0.16, 43.0]	14.4 [4.8, 100]	15.1 [1.5, 100]	7.69 [0.2, 100]
fPSA (ng/ml)	1.0 [0.2, 11.6]	1.8 [0.4, 23.2]	1.6 [0.3, 50.0]	1.2 [0.1, 50.0]
*ISUP Grade Group, n (%)*				
1	0 (0.0)	6 (9.5)	15 (17.6)	21 (4.4)
2	34 (10.2)	13 (20.6)	10 (11.8)	57 (11.9)
3	10 (3.0)	13 (20.6)	9 (10.6)	32 (6.7)
4	5 (1.5)	9 (14.3)	14 (16.5)	28 (5.8)
5	2 (0.6)	8 (12.7)	16 (18.8)	26 (5.4)
Positive but missing	18 (5.4)	0 (0.0)	2 (2.4)	20 (4.2)
No Cancer	263 (79.2)	14 (22.2)	19 (22.4)	296 (61.7)
*PI-RADS, n (%)*				
2	202 (60.8)	20 (31.7)	15 (17.6)	237 (49.4)
3	76 (22.9)	18 (28.6)	26 (30.6)	120 (25.0)
4	21 (6.3)	3 (4.8)	3 (3.5)	27 (5.6)
5	33 (10.0)	22 (34.9)	41 (48.3)	96 (20.0)
*Zone distribution of lesions in patients with PCa, n (%)*				
PZ	14 (20.3)	12 (25.0)	13 (20.6)	39 (21.7)
TZ	11 (15.9)	9 (18.8)	8 (12.7)	28 (15.6)
PZ and TZ	44 (63.8)	27 (56.3)	42 (66.7)	113 (62.8)
Median volume of lesions in patients with PCa, n (%)	1.1 [0.8, 2.2]	2.3 [1.1, 4.7]	3.2 [1.9, 7.1]	2.0 [1.0, 4.8]

PSA = prostate-specific antigen, SD = standard deviation, ISUP = International Society of Urological Pathology, PI-RADS = Prostate Imaging Reporting and Data System, PZ = peripheral zone, TZ = transition zone

Quantitative variables were given as the mean ± SD for normalized data and as the median [minimum, maximum] for nonnormalized data. The categorical variables are given as absolute frequency (relative frequency)

### Performance of Reader-AI and Reader-only

Table 3 shows the performance of Reader-AI and Reader-only. Reader-AI detected 302 suspected lesions. Among them, 206 (68.2%) were proved to be true positive lesions. Reader-only detected 304 suspected lesions, and 140 (46.5%) of them were proved to be true positive lesions. In terms of patient diagnosis, Reader-AI and Reader-only detected 168 (66.1%) and 159 (55.8%) true positive patients, respectively.

**Table 3:** Comparisons of Reader-AI and Reader-only to the reference standard on the lesion level and patient level

	Reference standards (lesion level)			Reference standards (patient level)		
	Positive	Negative	All	Positive	Negative	All
*Reader-AI*						
Positive	206	96	302	168	86	254
Negative	143	–	–	12	214	253
All	349	–	–	180	300	480
*Reader-only*						
Positive	140	164	304	159	126	285
Negative	209	–	–	21	174	195
All	349	–	–	180	300	480

Table 4 shows the comparison of readers' diagnostic performance under the two reading modes. On a lesion level, the mean sensitivity improved from 40.1% for Reader-only to 59.0% for Reader-AI (18.9% increased; 95% CI [11.5, 26.1]; *p* < 0.001). On patient level, the use of AI improved the mean specificity of radiologists from 57.7 to 71.7% (14.0% increase, 95% CI [6.4, 21.4]; *p* < 0.001) while preserving the sensitivity (88.3% for Reader-only and 93.9% for Reader-AI, *p* = 0.06).

**Table 4:** Comparisons of the clinically significant prostate cancer diagnosis performance between Reader-AI and Reader-only

Reading mode	Lesion level	Patient level	
	Sensitivity	Sensitivity	Specificity
Reader-only	40.1 [35.1, 45.3]	88.3 [82.8, 92.2]	57.7 [52.0, 63.1]
Reader-AI	59.0 [53.8, 64.1]	93.9 [89.4, 96.6]	71.7 [66.3, 76.5]
Difference	18.9 [11.5, 26.1]	5.6 [−0.4, 11.7]	14.0 [6.4, 21.4]
*p*	< .001*	0.060	< .001*

The 95% confidence intervals are shown in square brackets

*The difference was statistically significant (*p* < 0.05) according to the Chi-square test

### Reading and reporting times and diagnostic confidence

**Fig. 3:**
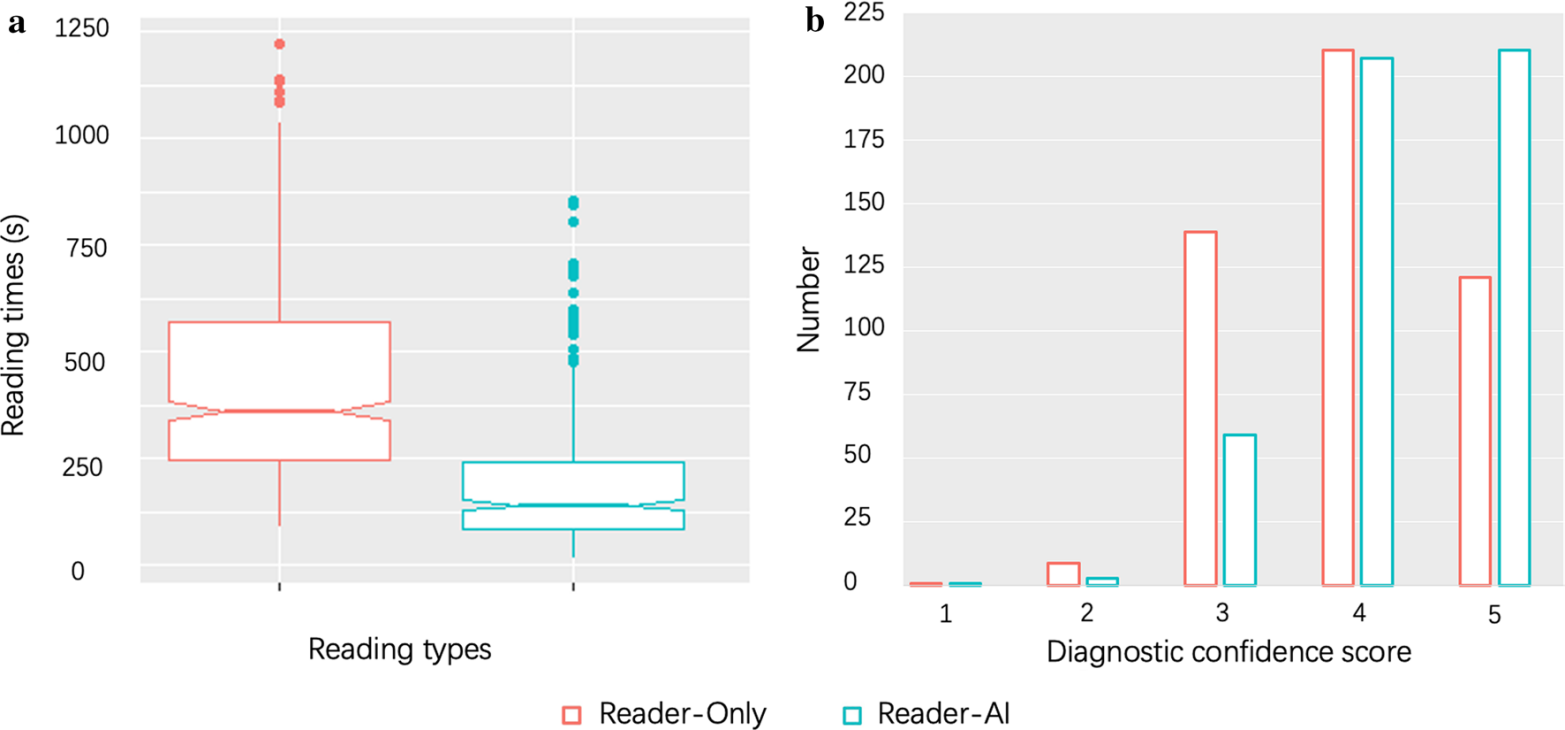
Notched box plot shows the changes in reading and reporting times (**a**). The bar plot shows the changes in diagnostic confidence (**b**)

The time records of the readings were missing in two cases. The median reading and reporting time of one case was reduced by 56.3% from 423 to 185 s (238-s decrease, 95% CI [219, 260]; *p* < 0.001) with the AI-aided procedure (Fig. 3A). The median diagnostic confidence was increased by 10.3% from 3.9 to 4.3 (0.4-score increase, 95% CI [0.3, 0.5]; *p* < 0.001) with the AI-aided procedure (Fig. 3B).

## Discussion

In this multicenter external validation study, the results showed that AI software substantially improved the lesion-level and patient-level specificity of the readers while preserving patient-level sensitivity in detecting MRI-visible csPCa. Meanwhile, with the help of AI, the radiologists reduced the mean reading and reporting time and increased the diagnostic confidence for diagnosis.

Recently, many studies [28] have emphasized the promising stand-alone AI performance for csPCa detection in mpMRI. Some researchers have further attempted to investigate how AI-assisted reading contributes to radiologists' interpretations of prostate MRIs. Several studies [26, 29–31] found that CAD-assisted reading improved sensitivity on patient level and/or lesion level, but specificity was sacrificed or not altered among radiologists. However, Niaf et al.'s study [32] showed that CAD could improve the classification specificity of lesions in the peripheral zone but not the sensitivity. The main finding in our study is that radiologists were significantly more sensitive to detecting MRI-visible csPCa foci with AI software assistance than they were without it, and their patient-level specificity also increased while not impairing patient-level sensitivity. The outcome discrepancy may be due to differences in external validation datasets, numbers of cases/lesions and parameters of the scanners. Of note, most previous studies provide results based on homogeneous data and propose the necessity of multicenter and multivendor research with an external dataset.

Similar to our study, Winkel et al. [33] conducted a study to validate the value of their prostate cancer CAD system for seven radiologists’ interpretations using a publicly available dataset in the PROSTATEx Challenge [34]. Their study used an external dataset, but the data were essentially homogeneous data from two different types of Siemens 3 T MR scanners, i.e., the MAGNETOM Trio and Skyra. The strength of our study is that the external data were collected from three different medical institutions. The mpMRI images were acquired using a total of 11 different MR devices with some variation in scan parameters. Thus, the data are very heterogeneous, which is a challenging task for AI algorithms.

In this retrospective study, the prevalence of prostate cancer was 37.5% for csPCa. Although not as good as prospective studies in real-world scenarios, the datasets we collected are a reasonable literature average, which is reflective of real-world datasets. However, data variations existed among the 3 hospitals. On the one hand, hospitals 2 and 3 have more advanced cases than hospital 1. On the other hand, most patients were examined at 3 T scanners, while approximately 5% of patients were examined at 1.5 T scanners. This may be related to different clinical protocols among these hospitals. Despite the above imbalances, it shows that our real-world dataset simulates a real-life clinical scenario and that the AI software can work with these imbalances, which lays a foundation for future prospective studies.

Several studies [30, 35] assessed the impact of a CAD system on less-experienced and experienced readers in mpMRI interpretation. The CAD system can significantly improve the performance of less-experienced readers to achieve expert-level performance. In our study, the 16 readers were from four hospitals, and their diagnostic experience varied widely (1- to 5-year experience). Each reader obtained reading cases from both their hospital and other hospitals. Therefore, for every reader, the data are heterogeneous and unfamiliar. Even so, the results of this study still show a significant improvement in diagnostic efficacy by using AI, indicating that the AI software used in this study has substantial generalization capability.

In this study, the patient-level sensitivity of Reader-only was 88.3%, and it increased to 93.9% in Reader-AI mode (*p* = 0.06). Even though the statistical significance of the difference in patient-level sensitivity was not achieved, we suppose it might be observed by increasing the sample size in a future study. Regarding the data, the sensitivity of Reader-only for MRI-visible csPCa lesion detection was 40.1%, and it was significantly increased to 59.0% (*p* < 0.001) with the Reader-AI procedure. At the same time, there is an improved patient-level specificity in the Reader-AI mode (57.7% vs. 71.7%, *p* < 0.001), which is beneficial to avoid unnecessary biopsies. Some studies indicate that the ability of humans to detect large lesions is usually sufficient on their own [36, 37], and AI software does not provide extra help for radiologists [26]. Thus, we excluded cases with prominent lesions as well as cases with advanced cancer, i.e., obvious extracapsular extension, diffuse pelvic lymph adenopathy and/or bone metastasis. The omission of these cases may lead to an underestimation of the diagnostic performance of Reader-only. Our findings suggest that the ability of radiologists to detect difficult lesions was significantly improved with the help of AI. Given the significant improvement in the detection of MRI-visible csPCa lesions by the Reader-AI mode, the AI-aided results have greater value in guiding the localization of biopsy.

In our study, a significant shortening in diagnostic time was observed in Reader-AI mode. Even so, our study's overall diagnostic time was longer than previously reported [29, 33]. In contrast to a previous study that only recorded the timing of prostate cancer detection, our reading procedure recorded the timing of the complete prostate interpretation report. The PI-RADS guidelines recommend a structured prostate report consisting of prostate volume measurement, detecting, measuring, characterizing and locating suspicious lesions, as well as other findings in the entire pelvis. Although our AI software only assisted in parts of the workflow, we can see that the efficiency of the overall report has been improved, indicating that embedding AI into structured reports is a good method to improve efficiency in clinical practice.

This study has obvious limitations. First, this study is a retrospective study, and a prospective study is of greater value. Second, the reference standard is based on cognitive fusion biopsy, which is subject to a higher risk of targeting errors than software fusion, so it is clearly a limitation. Thus, the annotations of the reference standard are possibly biased. And there is a lack of analysis of data that mismatch between MR images and pathology. The result obtained by using whole-mount step section pathology as the reference standard is more credible. Third, readers' experiences varied widely; thus, it was difficult to perform a stratification analysis. The consistency of all readers was also not analyzed because each case was evaluated by only one reader. Although there are 16 radiologists, each radiologist only read 30 cases. It would be better if all radiologists read all cases. Fourth, there is a gross imbalance in the datasets. Last but not least, we excluded data with poor image quality that should also be analyzed in our daily work.

To conclude, this multicenter, self-crossover-controlled study showed that AI software, when tested in high-quality, real-world datasets, improves the diagnostic performance of radiologists by reducing the detection of false positive patients and also improving reading and reporting times and diagnostic confidence.

## Supplementary Information

**Additional file 1:** Information on the development and performance of the AI software.

## Data Availability

The datasets used and analyzed during the current study are available from the corresponding author on reasonable request.

## Abbreviations

CAD: Computer-aided detection
CI: Confidence interval
csPCa: Clinically significant prostate cancer
ISUP: International Society of Urological Pathology
mpMRI: Multiparametric magnetic resonance imaging
PI-RADS: Prostate Imaging Reporting and Data System
PSA: Prostate-specific antigen
TRUS: Transrectal ultrasound
