# Supplementary material for: A multicenter study of artificial intelligence-aided software for detecting visible clinically significant prostate cancer on mpMRI
Source: Insights Imaging. 2023 Apr 30;14:72. doi: 10.1186/s13244-023-01421-w (PMC10149551; doi:10.1186/s13244-023-01421-w)

A multicenter study of artificial intelligence-aided software for detecting visible clinically significant prostate cancer on mpMRI

(i) MRI sequence classification

Data enrollment

The mpMRI images were retrospectively collected from 1,086 patients (1,153 mpMRI examinations) studied from July 28, 2009, to Nov. 26, 2021. After importing the anonymized data, the DICOM data were converted to Nifty format using dicom2nii.py (Python 3.5) to obtain the image data. First, the DICOM data were split into multiple scan sequences for one MR examination. Individual sequences with more than 15 slices were included in the study. Then, each sequence was further split into an image group. The images with the same acquisition parameters and the same spatial location were split into one image group. The diffusion-weighted imaging (DWI) sequence was grouped by b-value, for example, a DWI sequence with 3 b-values was split into 3 independent image groups, with each image group having only one unique b-value. A total of 5151 images from five image types were eventually classified, including 1) DWI_High (b value ≥ 500 s/mm2, n=1045), 2) DWI_Low (b value ≤ 100 s/mm2, n=1012), 3) apparent diffusion coefficient (ADC) map (n=906), 4) T2-weighted imaging_nan (T2WI_nan) (non fat-sat T2WI, n=1000), and 5) T2WI_fs (fat-sat T2WI, n=1188). The T1-weighted imaging (T1WI) and dynamic enhancement (DCE) images were scanned but excluded from the study.

MR scanners and imaging protocols

The mpMRI images were obtained from 15 MR scanners of four vendors. The transmit coils were body coils, and the receiver coils were phased array coils. No endorectal coil was used. Information on the MR scanners and image types is shown in Table S1.

Development of deep-learning model

A modified Med3D network (Figure S1) was retrained to classify the sequences of prostate

mpMRI. The classification efficiency was evaluated by the confusion matrix.

Results

The confusion matrixes of the prediction results in different datasets are shown in Figure S2. The corresponding prediction efficacies of the image classification model in different datasets are shown in Table S2. The prediction accuracies in the training, validation, and testing datasets were 0.992~1.000, 0.989~1.000, and 0.995~1.000, respectively.

(ii) Prostate gland segmentation and measurement

Data enrollment

The mpMRI images were retrospectively collected from 2,673 patients (2,849 mpMRI examinations) studied from July 28, 2009, to Nov. 26, 2021.

After importing the anonymized data, the DICOM data were converted to nifty format using dicom2nii.py (Python 3.5). The ADC maps (n=2320) were calculated from the DWI sequence with high and low b-values. Conventional T2WI and fat saturation T2WI (fat-sat T2WI) (n=3654) were selected.

MR scanners and imaging protocols

The mpMRI images were obtained from 19 MR scanners of four vendors. The transmit coils were body coils, and the receiver coils were phased array coils. No endorectal coil was used. Information on the MR scanners and image types is shown in Table S3.

Development of deep-learning model

The ground truth of prostate gland was manually outlined by two experts with more than 5 years of experience. The ADC and T2WI images were resized to 64×256×224 (z, y, x) pixels and were taken as the input of the network. We augmented the data in the training set by random rotation (rotation angle within 10°), adding random noise, and parallel translation at a range of [(-0.1; 0.1); (-0.1; 0.1)] pixels.

A preliminary experiment has been published [1]. We use the classic U-Net [2] framework, which enables accurate pixelwise prediction by combining spatial and contextual information in a network architecture comprising convolutional layers. All the training and experiments were conducted on a personal computer equipped with an Intel Core i5 3.2 GHz CPU with 16 GB main memory and an NVIDIA GTX1060 GPU. The proposed deep-learning network was implemented using the Keras open-source deep-learning library, and TensorFlow was chosen as a backend deep-learning engine. The learning rate was set as 0.0001, and the U-Net models were trained for up to 400 iterations.

Results

Dice similarity coefficient (DSC), Jacard index, volumetric similarity (VS), Hausdorf distance (HD), and Average distance (AD) were used to compare the model and manual segmentation results. The right and left (RL) diameter, anterior and posterior (AP) diameter, and superior and inferior (SI) diameter of the prostate gland were automatically measured using the algorithm rule of the minimum volume bounding box (Figure S3).

The DSC, Jacard index, VS, HD, and AD in different datasets are shown in Table S4 and Figure S4. The segmentation metrics of the T2WI were superior to those of the ADC map in all datasets (all $P<0.001$). Bland–Altman analysis of the measured values of the prostate gland, including RL diameter, AP diameter, SI diameter, volume, and signal intensity, are shown in Table S5 and Figure S5. The differences between the manual label and the predicted label to their means were -2.058% to 4.257%.

(iii) Prostate zonal anatomy segmentation

Sextant locations

First, the prostate gland is segmented by the established model (refer to part ii). For sextant location, the prostatic gland was then trisected to obtain the base, mid-gland, and apex in the longitudinal axis direction. It was bisected to divide the prostate gland into left and right parts

in the horizontal axis direction. Thus, the sextants were automatically generated (Figure S6). When one sextant overlaps with a lesion, it is considered a cancer sextant; otherwise, it is considered a non-cancer sextant.

Second, for the anatomic zone locations, we developed an anatomic regional model to segment the peripheral zone (PZ), transition zone (TZ), central zone (CZ), anterior fibromuscular stroma (AFMS), urethra (URE), left seminal vesicle (LS) and right seminal vesicle (RS) (Figure S7).

Data enrollment

The mpMRI images were retrospectively collected from 1225 patients from August 29, 2012, to Nov. 26, 2021. After importing the anonymized data, the DICOM data were converted to nifty format using dicom2nii.py (Python 3.5). T2WI images were used for the development of the prostate zonal anatomy segmentation model.

MR scanners and imaging protocols

The T2WI images were obtained from 17 MR scanners of four vendors. The transmit coils were body coils, and the receiver coils were phased array coils. No endorectal coil was used. Information on the MR scanning protocols is shown in Table S6.

Development of deep-learning model

The T2WI images were resized to 64×256×224 (z, y, x) pixels and were taken as the input of the network. We augmented the data in the training set by random rotation (rotation angle within 10°), adding random noise, and parallel translation at a range of [(-0.1; 0.1); (-0.1; 0.1)] pixels. A total of 1225 images was randomly split into 80% training, 10% validation, and 10% test sets. A 3D U-Net segmentation framework [2] is used for prostate anatomic zone segmentation. The model took the T2 weight image as input. All the training processes were performed using the GPU NVIDIA Tesla P100 16G. The algorithm was coded by Python 3.6, PyTorch 0.4.1, OpenCV 3.4.0.12, Numpy 1.16.2, and SimpleITK 1.2.0. The batch size was set as 10. The networks were trained for a total of 300 epochs. Adam was employed as an

optimizer to minimize loss with a learning rate of 0.0001 and a binary cross-entropy loss function.

Result

The DSC, Jacard index, VS, HD, and AD in different datasets are shown in Table S7. The median metrics in the training, validation, and test dataset showed statistically significant differences (P<0.001). When one zone overlaps with a lesion, it is considered a cancer zone; otherwise, it is considered a non-cancer zone.

(iv) CsPCa foci segmentation and measurement

Data enrollment

The mpMRI images were retrospectively collected from 2105 patients from Jan. 10, 2014, to Nov. 26, 2021, including 1368 patients with PCa and 737 patients without PCa. After importing the anonymized data, the DICOM data were converted to nifty format using dicom2nii.py (Python 3.5). The ADC maps were calculated from the DWI sequence with high and low b-values.

MR scanners and imaging protocols

The mpMRI images were obtained from 18 MR scanners of four vendors. The transmit coils were body coils, and the receiver coils were phased array coils. No endorectal coil was used. The DWI and its corresponding ADC map were selected to develop the deep-learning model. Information on the MR scanning protocols is shown in Table S8.

Development of deep-learning model

The prostate gland region was automatically pre-segmented using model (refer to part ii) previously developed in our institution. Once the prostate region was identified, the images were cropped to a patch of size. All the images were unified and cropped to 64×64×64 (z, y, x) and then normalized of pixel intensity to an interval of [0,1]. We augmented the data in the

training set by random rotation (rotation angle within 10°), adding random noise, and parallel translation at a range of [(-0.1; 0.1); (-0.1; 0.1)]. The data set was randomly split into 80% training, 10% validation, and 10% internal test sets.

A 3D U-net [2] segmentation framework is used for prostate cancer foci segmentation. The AI model took the combination of DWI and ADC maps as input. All the training processes were performed using the GPU NVIDIA Tesla P100 16G. The algorithm was coded by Python 3.6, PyTorch 0.4.1, OpenCV 3.4.0.12, Numpy 1.16.2, and SimpleITK 1.2.0. The batch size was set as 20. The networks were trained for a total of 1000 epochs. Adam was employed as an optimizer to minimize loss with a learning rate of 0.0001 and a binary cross-entropy loss function. Based on the current capabilities of mpMRI [3], csPCa greater than or equal to 0.5 cc in volume may be detected. To minimize the influence of very small tumor foci, the model outputs were filtered with a threshold value of 0.5 cc. The max diameter of csPCa lesions was automatically measured by using the algorithm of the minimum volume bounding box. The volume of csPCa was calculated according to the number of pixels in the remaining domains.

Results

The DSC, Jacard index, VS, HD, and AD in different datasets are shown in Table S9 and Figure S8. The median DSC values were 0.93, 0.81, and 0.80 in the training, validation, and test dataset ($P<0.001$). The measurements of the prostate cancer foci, including lesion number, RL diameter, AP diameter, SI diameter, volume, and signal intensity, are shown in Table S10. Bland–Altman analysis of the measured values of the prostate cancer foci, including RL diameter, AP diameter, SI diameter, volume, and signal intensity, are shown in Table S11 and Figure S9.

References

1. Zhu Y, Wei R, Gao G, Ding L, Zhang X, Wang X, Zhang J (2019) Fully automatic segmentation on prostate MR images based on cascaded fully convolution network. J Magn Reson Imaging 49(4):1149-1156. doi: 10.1002/jmri.26337

2. Çiçek Ö, Abdulkadir A, Lienkamp SS, Brox T, Ronneberger O (2016) 3D U-Net: Learning Dense Volumetric Segmentation from Sparse Annotation. In: Ourselin S, Joskowicz L, Sabuncu MR, Unal G, Wells W, eds. Medical Image Computing and Computer-Assisted Intervention – MICCAI 2016. Cham: Springer International Publishing, p. 424-432

3. Turkbey B, Rosenkrantz AB, Haider MA, Padhani AR, Villeirs G, Macura KJ, Tempany CM, Choyke PL, Cornud F, Margolis DJ, Thoeny HC, Verma S, Barentsz J, Weinreb JC (2019) Prostate Imaging Reporting and Data System Version 2.1: 2019 Update of Prostate Imaging Reporting and Data System Version 2. Eur Urol 76(3):340-351. doi: 10.1016/j.eururo.2019.02.033

Table S1. Information on the MR scanners and the image types.

	Overall (n=5151)	Training (n=4122)	Validation (n=513)	Testing (n=516)	*P* value
**Age (yrs)**					
Median [Q1, Q3]	71.0 [65.0, 76.0]	71.0 [65.0, 76.0]	71.0 [66.0, 77.0]	71.0 [65.0, 76.0]	0.461
**Image Type**					
ADC	906 (17.6%)	730 (17.7%)	86 (16.8%)	90 (17.4%)	1.000
DWI_High	1045 (20.3%)	835 (20.3%)	105 (20.5%)	105 (20.3%)	
DWI_Low	1012 (19.6%)	808 (19.6%)	102 (19.9%)	102 (19.8%)	
T2WI_Fs	1188 (23.1%)	950 (23.0%)	120 (23.4%)	118 (22.9%)	
T2WI_nan	1000 (19.4%)	799 (19.4%)	100 (19.5%)	101 (19.6%)	
**Magnetic Field**					
1.5 T	657 (12.8%)	523 (12.7%)	59 (11.5%)	75 (14.5%)	0.331
3.0 T	4494 (87.2%)	3599 (87.3%)	454 (88.5%)	441 (85.5%)	
**Manufacture**					
GE Medical Systems	2635 (51.2%)	2100 (50.9%)	253 (49.3%)	282 (54.7%)	0.495
Philips Medical Systems	491 (9.5%)	397 (9.6%)	50 (9.7%)	44 (8.5%)	
SIEMENS	2025 (39.3%)	1625 (39.4%)	210 (40.9%)	190 (36.8%)	
**Station Name**					
AWP145938	597 (11.6%)	468 (11.4%)	73 (14.2%)	56 (10.9%)	0.169
AWP152194	119 (2.3%)	96 (2.3%)	12 (2.3%)	11 (2.1%)	
AWP166059	194 (3.8%)	164 (4.0%)	17 (3.3%)	13 (2.5%)	
AWP174090	8 (0.2%)	5 (0.1%)	0 (0%)	3 (0.6%)	
AWP39300	6 (0.1%)	5 (0.1%)	0 (0%)	1 (0.2%)	
DVMRDVMR	1172 (22.8%)	939 (22.8%)	124 (24.2%)	109 (21.1%)	
GEHC	1023 (19.9%)	821 (19.9%)	87 (17.0%)	115 (22.3%)	
GEHCGEHC	440 (8.5%)	340 (8.2%)	42 (8.2%)	58 (11.2%)	
MRC35207	696 (13.5%)	567 (13.8%)	69 (13.5%)	60 (11.6%)	
MRC40764	387 (7.5%)	306 (7.4%)	37 (7.2%)	44 (8.5%)	
MRSUZTB03A	57 (1.1%)	49 (1.2%)	4 (0.8%)	4 (0.8%)	
PHILIPS-8FA1B4E	72 (1.4%)	62 (1.5%)	5 (1.0%)	5 (1.0%)	
PHILIPS-CB0GKAC	12 (0.2%)	9 (0.2%)	0 (0%)	3 (0.6%)	
PHILIPS-DSALI1J	156 (3.0%)	124 (3.0%)	17 (3.3%)	15 (2.9%)	
PHILIPS-NK6RG9A	194 (3.8%)	153 (3.7%)	24 (4.7%)	17 (3.3%)	

Table S2. Prediction efficacies of the image classification model in different datasets.

Image Type	Image Number	Accuracy	Sensitivity	Specificity	PPV	NPV	F1	Kappa	Prevalence	Detection Rate	Detection Prevalence
**Training**											
ADC	718	1.000	1.000	1.000	0.999	1.000	0.999	0.999	0.174	0.174	0.174
DWI_High	849	0.996	0.994	0.998	0.991	0.998	0.992	0.990	0.206	0.205	0.207
DWI_Low	815	0.992	0.987	0.998	0.991	0.997	0.989	0.986	0.198	0.195	0.197
T2WI_Fs	957	0.998	0.997	0.999	0.998	0.999	0.997	0.997	0.232	0.231	0.232
T2WI_nan	783	1.000	1.000	1.000	0.999	1.000	0.999	0.999	0.190	0.190	0.190
**Validation**											
ADC	96	1.000	1.000	1.000	1.000	1.000	1.000	1.000	0.187	0.187	0.187
DWI_High	93	0.989	0.978	1.000	1.000	0.995	0.989	0.987	0.181	0.177	0.177
DWI_Low	101	0.998	1.000	0.995	0.981	1.000	0.990	0.988	0.197	0.197	0.201
T2WI_Fs	107	1.000	1.000	1.000	1.000	1.000	1.000	1.000	0.209	0.209	0.209
T2WI_nan	116	1.000	1.000	1.000	1.000	1.000	1.000	1.000	0.226	0.226	0.226
**Testing**											
ADC	92	1.000	1.000	1.000	1.000	1.000	1.000	1.000	0.178	0.178	0.178
DWI_High	103	0.995	0.990	1.000	1.000	0.998	0.995	0.994	0.200	0.198	0.198
DWI_Low	96	0.999	1.000	0.998	0.990	1.000	0.995	0.994	0.186	0.186	0.188
T2WI_Fs	124	1.000	1.000	1.000	1.000	1.000	1.000	1.000	0.240	0.240	0.240
T2WI_nan	101	1.000	1.000	1.000	1.000	1.000	1.000	1.000	0.196	0.196	0.196

ADC, apparent diffusion coefficient; T2WI, T2-weighted imaging; DWI, diffusion-weighted imaging; Fs, fat-sat; PPV, positive predictive value; NPV, negative predictive value.

Table S3. Information on the MR scanners and the image types.

	Overall (n=5974)	Training (n=4780)	Validation (n=601)	Testing (n=593)	*P* Value
**Age (yrs)**					0.286
Median [Q1, Q3]	70.0 [64.0, 76.0]	70.0 [64.0, 76.0]	70.0 [64.0, 76.0]	70.0 [63.0, 75.0]	
**Magnetic Field**					0.498
1.5T	1034 (17.3%)	841 (17.6%)	96 (16.0%)	97 (16.4%)	
3.0T	4940 (82.7%)	3939 (82.4%)	505 (84.0%)	496 (83.6%)	
**Image Type**					0.345
ADC	2320 (38.8%)	1873 (39.2%)	217 (36.1%)	230 (38.8%)	
T2WI	3654 (61.2%)	2907 (60.8%)	384 (63.9%)	363 (61.2%)	
**Manufacture**					0.792
GE MEDICAL SYSTEMS	3243 (54.3%)	2599 (54.4%)	316 (52.6%)	328 (55.3%)	
Philips Medical Systems	810 (13.6%)	637 (13.3%)	91 (15.1%)	82 (13.8%)	
SIEMENS	1695 (28.4%)	1368 (28.6%)	168 (28.0%)	159 (26.8%)	
UIH	226 (3.8%)	176 (3.7%)	26 (4.3%)	24 (4.0%)	
**Model Name**					0.874
Achieva	150 (2.5%)	123 (2.6%)	17 (2.8%)	10 (1.7%)	
Ingenia	583 (9.8%)	456 (9.5%)	64 (10.6%)	63 (10.6%)	
Ingenia CX	3 (0.1%)	2 (0.0%)	1 (0.2%)	0 (0%)	
Discovery MR750	2753 (46.1%)	2204 (46.1%)	276 (45.9%)	273 (46.0%)	
Discovery MR750w	304 (5.1%)	250 (5.2%)	21 (3.5%)	33 (5.6%)	
Signa EXCITE	173 (2.9%)	135 (2.8%)	16 (2.7%)	22 (3.7%)	
Signa HDxt	11 (0.2%)	9 (0.2%)	2 (0.3%)	0 (0%)	
Signa Premier	2 (0.0%)	1 (0.0%)	1 (0.2%)	0 (0%)	
Aera	889 (14.9%)	724 (15.1%)	81 (13.5%)	84 (14.2%)	
Amira	4 (0.1%)	3 (0.1%)	1 (0.2%)	0 (0%)	
Essenza	2 (0.0%)	2 (0.0%)	0 (0%)	0 (0%)	
Multiva	74 (1.2%)	56 (1.2%)	9 (1.5%)	9 (1.5%)	
Prisma	127 (2.1%)	99 (2.1%)	16 (2.7%)	12 (2.0%)	
Skyra	188 (3.1%)	156 (3.3%)	18 (3.0%)	14 (2.4%)	
TrioTim	348 (5.8%)	273 (5.7%)	39 (6.5%)	36 (6.1%)	
Verio	137 (2.3%)	111 (2.3%)	13 (2.2%)	13 (2.2%)	
uMR 790	226 (3.8%)	176 (3.7%)	26 (4.3%)	24 (4.0%)	

ADC, apparent diffusion coefficient; T2WI, T2-weighted imaging.

Table S4. Segmentation metrics in different datasets.

	Overall		Training		Validation		Testing	
	ADC (n=2320)	T2WI (n=3654)	ADC (n=1873)	T2WI (n=2907)	ADC (n=217)	T2WI (n=384)	ADC (n=230)	T2WI (n=363)
DSC	0.921 (0.0337)	0.937 (0.0322)	0.925 (0.0279)	0.940 (0.0270)	0.902 (0.0491)	0.922 (0.0445)	0.900 (0.0446)	0.923 (0.0449)
JACRD	0.854 (0.0549)	0.883 (0.0537)	0.862 (0.0467)	0.889 (0.0458)	0.825 (0.0755)	0.857 (0.0717)	0.822 (0.0704)	0.861 (0.0726)
VS	0.970 (0.0303)	0.979 (0.0245)	0.974 (0.0236)	0.981 (0.0200)	0.953 (0.0478)	0.967 (0.0370)	0.955 (0.0449)	0.971 (0.0339)
HD	6.460 (3.150)	5.880 (2.830)	6.20 (2.720)	5.640 (2.490)	7.450 (4.700)	6.780 (3.730)	7.700 (3.990)	6.840 (3.730)
AD	0.140 (0.149)	0.168 (4.01)	0.122 (0.0962)	0.172 (4.49)	0.213 (0.293)	0.152 (0.196)	0.214 (0.233)	0.149 (0.186)

DSC, Dice similarity coefficient; JACRD, Jacard index; VS, volumetric similarity; HD, Hausdorf distance; AD, Average distance; T2WI, T2-weighted imaging; ADC, apparent diffusion coefficient.

Table S5. Bland–Altman analysis of the measured values of the prostate gland.

	RL Diameter (mm)	AP Diameter (mm)	SI Diameter (mm)	Volume (cm^3)	Signal Intensity
Means of label and plabel	56.025	59.670	63.655	108.623	56.025
Differences	2.230	2.540	-1.310	-0.924	2.230
Means/differences proportion	3.980	4.257	-2.058	-0.851	3.980
Means of label	57.140	60.940	63.000	108.160	57.140
Means of plabel	54.910	58.400	64.310	109.085	54.910
Bias of the label and plabel	0.733	1.094	-1.180	-0.623	0.733
Bias Upper CI	0.800	1.172	-1.063	-0.477	0.800
Bias Lower CI	0.665	1.017	-1.298	-0.769	0.665
Bias Std Dev	2.679	3.057	4.643	5.756	2.679
Bias Standard Error	0.035	0.040	0.060	0.745	0.035
LOA Standard Error	0.059	0.068	0.103	0.127	0.059
Upper LOA	5.984	7.085	7.919	10.659	5.984
Upper LOA_upperCI	6.100	7.218	8.120	10.908	6.100
Upper LOA_lowerCI	5.868	6.953	7.718	10.409	5.868
Lower LOA	-4.519	-4.897	-10.280	-11.904	-4.519
Lower LOA_upperCI	-4.403	-4.764	-10.078	-11.655	-4.403
Lower LOA_lowerCI	-4.635	-5.029	-10.481	-12.154	-4.635
Regression fixed slope	0.076	0.071	0.032	0.023	0.076
Regression fixed intercept	-3.100	-2.100	-2.700	-1.900	-3.100

LOA, limits of agreement; CI, confidence interval; RL, right and left; AP, anterior and posterior; SI, superior and inferior.

Table S6. Scanning protocols of the T2WI.

	Overall (N=1225)	train (N=973)	validate (N=99)	test (N=153)	*P* value
**Magnetic Field**					
1.5T	271 (22.1%)	213 (21.9%)	14 (14.1%)	44 (28.8%)	0.022
3.0T	954 (77.9%)	760 (78.1%)	85 (85.9%)	109 (71.2%)	
**Manufacture**					
GE MEDICAL SYSTEMS	665 (54.3%)	540 (55.5%)	65 (65.7%)	60 (39.2%)	<0.001
Philips Medical Systems	155 (12.7%)	111 (11.4%)	10 (10.1%)	34 (22.2%)	
SIEMENS	365 (29.8%)	289 (29.7%)	20 (20.2%)	56 (36.6%)	
UIH	40 (3.3%)	33 (3.4%)	4 (4.0%)	3 (2.0%)	
**Model Name**					
Achieva	26 (2.1%)	20 (2.1%)	3 (3.0%)	3 (2.0%)	0.011
Aera	239 (19.5%)	192 (19.7%)	8 (8.1%)	39 (25.5%)	
Amira	1 (0.1%)	1 (0.1%)	0 (0%)	0 (0%)	
DISCOVERY MR750	547 (44.7%)	446 (45.8%)	54 (54.5%)	47 (30.7%)	
DISCOVERY MR750w	78 (6.4%)	64 (6.6%)	5 (5.1%)	9 (5.9%)	
Ingenia	111 (9.1%)	79 (8.1%)	5 (5.1%)	27 (17.6%)	
Ingenia CX	1 (0.1%)	1 (0.1%)	0 (0%)	0 (0%)	
MAGNETOM_ESSENZA	1 (0.1%)	1 (0.1%)	0 (0%)	0 (0%)	
Multiva	17 (1.4%)	11 (1.1%)	2 (2.0%)	4 (2.6%)	
Prisma	10 (0.8%)	7 (0.7%)	1 (1.0%)	2 (1.3%)	
SIGNA EXCITE	36 (2.9%)	28 (2.9%)	5 (5.1%)	3 (2.0%)	
Signa HDxt	3 (0.2%)	1 (0.1%)	1 (1.0%)	1 (0.7%)	
SIGNA Premier	1 (0.1%)	1 (0.1%)	0 (0%)	0 (0%)	
Skyra	34 (2.8%)	28 (2.9%)	3 (3.0%)	3 (2.0%)	
TrioTim	41 (3.3%)	35 (3.6%)	2 (2.0%)	4 (2.6%)	
uMR 790	40 (3.3%)	33 (3.4%)	4 (4.0%)	3 (2.0%)	
Verio	39 (3.2%)	25 (2.6%)	6 (6.1%)	8 (5.2%)	
**FatSat**					
fs	87 (7.1%)	67 (6.9%)	10 (10.1%)	10 (6.5%)	0.474
non-fs	1138 (92.9%)	906 (93.1%)	89 (89.9%)	143 (93.5%)	
Repetition Time (ms)					
Median [Q1, Q3]	3560 [3040, 3880]	3460 [3040, 3850]	3560 [3070, 3790]	3730 [3000, 4200]	0.296
**Echo Time (ms)**					
Median [Q1, Q3]	92.9 [87.5, 112]	92.2 [87.4, 110]	90.3 [87.4, 103]	99.0 [88.0, 115]	0.053
**Pixel Bandwidth (Hz)**					
Median [Q1, Q3]	163 [163, 200]	163 [163, 200]	163 [122, 188]	200 [160, 218]	<0.001
**Flip Angle**					
Median [Q1, Q3]	111 [111, 140]	111 [111, 140]	111 [111, 111]	111 [111, 150]	0.366
**Reconstruction Diameter**					

Median [Q1, Q3]	240 [200, 240]	240 [200, 240]	240 [200, 240]	220 [200, 240]	0.007
**Slice Thickness (mm)**					
Median [Q1, Q3]	4.00 [3.50, 4.00]	4.00 [3.50, 4.00]	4.00 [3.40, 4.00]	4.00 [3.50, 4.00]	0.443
**Slice Spacing (mm)**					
Median [Q1, Q3]	4.00 [4.00, 4.00]	4.00 [4.00, 4.00]	4.00 [4.00, 4.00]	4.00 [3.60, 4.00]	0.062
**Pixel Spacing (mm)**					
Median [Q1, Q3]	0.469 [0.469, 0.577]	0.469 [0.469, 0.625]	0.469 [0.417, 0.469]	0.469 [0.344, 0.625]	0.054

Table S7. Segmentation metrics of the model

	Overall (N=1225)	train (N=979)	validate (N=123)	test (N=123)	P Value
**AFS**					
**DICE**					
Median [Min, Max]	0.790 [0, 0.920]	0.800 [0, 0.920]	0.710 [0, 0.890]	0.690 [0.0300, 0.860]	<0.001
Missing	11 (0.9%)	8 (0.8%)	1 (0.8%)	2 (1.6%)	
**JACRD**					
Median [Min, Max]	0.650 [0, 0.850]	0.670 [0, 0.850]	0.550 [0, 0.800]	0.530 [0.020, 0.760]	<0.001
Missing	11 (0.9%)	8 (0.8%)	1 (0.8%)	2 (1.6%)	
**VOLSMTY**					
Median [Min, Max]	0.930 [0.0300, 1.00]	0.940 [0.0300, 1.00]	0.885 [0.230, 1.00]	0.890 [0.140, 1.00]	<0.001
Missing	11 (0.9%)	8 (0.8%)	1 (0.8%)	2 (1.6%)	
**HDRFDST**					
Median [Min, Max]	5.05 [1.56, 50.3]	4.77 [1.56, 50.3]	6.89 [2.21, 47.0]	6.64 [2.50, 48.6]	<0.001
Missing	11 (0.9%)	8 (0.8%)	1 (0.8%)	2 (1.6%)	
**AVGDIST**					
Median [Min, Max]	0.260 [0.0900, 25.3]	0.230 [0.090, 25.3]	0.410 [0.100, 9.58]	0.450 [0.130, 3.73]	<0.001
Missing	11 (0.9%)	8 (0.8%)	1 (0.8%)	2 (1.6%)	
**PZ**					
**DICE**					
Median [Min, Max]	0.870 [0, 0.960]	0.88 [0, 0.96]	0.84 [0.48, 0.92]	0.840 [0.390, 0.930]	<0.001
Missing	1 (0.1%)	1 (0.1%)	0 (0%)	0 (0%)	
**JACRD**					
Median [Min, Max]	0.770 [0, 0.920]	0.780 [0, 0.920]	0.720 [0.31, 0.86]	0.720 [0.240, 0.870]	
Missing	1 (0.1%)	1 (0.1%)	0 (0%)	0 (0%)	
**VOLSMTY**					
Median [Min, Max]	0.970 [0.100, 1.00]	0.970 [0.100, 1.00]	0.95 [0.61, 1.00]	0.960 [0.530, 1.00]	<0.001
Missing	1 (0.1%)	1 (0.1%)	0 (0%)	0 (0%)	
**HDRFDST**					
Median [Min, Max]	7.55 [2.50, 50.2]	7.20 [2.50, 43.7]	8.91 [2.58, 50.2]	8.11 [3.85, 44.3]	<0.001
Missing	1 (0.1%)	1 (0.1%)	0 (0%)	0 (0%)	
**AVGDIST**					
Median [Min, Max]	0.160 [0.0500, 19.5]	0.150 [0.0500, 19.5]	0.240 [0.080, 2.05]	0.240 [0.080, 4.43]	<0.001
Missing	1 (0.1%)	1 (0.1%)	0 (0%)	0 (0%)	
**CZ**					
**DICE**					
Median [Min, Max]	0.810 [0, 0.930]	0.820 [0.410, 0.930]	0.650 [0.05, 0.87]	0.630 [0, 0.900]	<0.001
Missing	6 (0.5%)	6 (0.6%)	0 (0%)	0 (0%)	
**JACRD**					
Median [Min, Max]	0.680 [0, 0.880]	0.700 [0.260, 0.880]	0.480 [0.03, 0.770]	0.460 [0, 0.810]	<0.001

Missing	6 (0.5%)	6 (0.6%)	0 (0%)	0 (0%)	
**VOLSMTY**					
Median [Min, Max]	0.920 [0.170, 1.00]	0.930 [0.490, 1.00]	0.88 [0.180, 1.00]	0.880 [0.170, 1.00]	<0.001
Missing	6 (0.5%)	6 (0.6%)	0 (0%)	0 (0%)	
**HDRFDST**					
Median [Min, Max]	4.60 [2.00, 45.5]	4.29 [2.00, 34.1]	6.53 [2.80, 45.5]	6.50 [2.73, 33.0]	<0.001
Missing	6 (0.5%)	6 (0.6%)	0 (0%)	0 (0%)	
**AVGDIST**					
Median [Min, Max]	0.240 [0.0600, 9.09]	0.220 [0.060, 3.89]	0.600 [0.140, 8.41]	0.610 [0.120, 9.09]	<0.001
Missing	6 (0.5%)	6 (0.6%)	0 (0%)	0 (0%)	
**TZ**					
**DICE**					
Median [Min, Max]	0.930 [0.610, 0.970]	0.940 [0.720, 0.970]	0.910 [0.610, 0.970]	0.920 [0.700, 0.970]	<0.001
Missing	1 (0.1%)	1 (0.1%)	0 (0%)	0 (0%)	
**JACRD**					
Median [Min, Max]	0.870 [0.440, 0.950]	0.880 [0.560, 0.950]	0.830 [0.44, 0.940]	0.850 [0.540, 0.940]	<0.001
Missing	1 (0.1%)	1 (0.1%)	0 (0%)	0 (0%)	
**VOLSMTY**					
Median [Min, Max]	0.980 [0.770, 1.00]	0.990 [0.840, 1.00]	0.970 [0.770, 1.00]	0.970 [0.830, 1.00]	<0.001
Missing	1 (0.1%)	1 (0.1%)	0 (0%)	0 (0%)	
**HDRFDST**					
Median [Min, Max]	4.59 [2.34, 38.3]	4.46 [2.34, 34.0]	5.33 [2.47, 38.3]	4.91 [2.72, 19.4]	<0.001
Missing	1 (0.1%)	1 (0.1%)	0 (0%)	0 (0%)	
**AVGDIST**					
Median [Min, Max]	0.080 [0.020, 1.07]	0.080 [0.020, 0.890]	0.130 [0.03, 1.07]	0.110 [0.0300, 0.730]	<0.001
Missing	1 (0.1%)	1 (0.1%)	0 (0%)	0 (0%)	
**URE**					
**DICE**					
Median [Min, Max]	0.910 [0, 0.980]	0.920 [0.520, 0.980]	0.830 [0, 0.960]	0.830 [0.490, 0.960]	<0.001
Missing	8 (0.7%)	5 (0.5%)	1 (0.8%)	2 (1.6%)	
**JACRD**					
Median [Min, Max]	0.830 [0, 0.960]	0.840 [0.350, 0.960]	0.700 [0, 0.930]	0.700 [0.320, 0.930]	<0.001
Missing	8 (0.7%)	5 (0.5%)	1 (0.8%)	2 (1.6%)	
**VOLSMTY**					
Median [Min, Max]	0.940 [0.0800, 1.00]	0.950 [0.550, 1.00]	0.890 [0.080, 1.00]	0.900 [0.490, 1.00]	<0.001
Missing	8 (0.7%)	5 (0.5%)	1 (0.8%)	2 (1.6%)	
**HDRFDST**					
Median [Min, Max]	1.88 [0.780, 49.5]	1.75 [0.780, 49.5]	3.31 [0.940, 17.1]	3.13 [0.780, 33.8]	<0.001
Missing	8 (0.7%)	5 (0.5%)	1 (0.8%)	2 (1.6%)	
**AVGDIST**					
Median [Min, Max]	0.0900 [0.020, 723]	0.080 [0.020, 1.18]	0.220 [0.04, 723]	0.200 [0.03, 1.13]	<0.001
Missing	8 (0.7%)	5 (0.5%)	1 (0.8%)	2 (1.6%)	

**RS**					
**DICE**					
Median [Min, Max]	0.920 [0, 0.970]	0.930 [0, 0.970]	0.900 [0.710, 0.97]	0.900 [0, 0.970]	<0.001
Missing	1 (0.1%)	1 (0.1%)	0 (0%)	0 (0%)	
**JACRD**					
Median [Min, Max]	0.860 [0, 0.940]	0.860 [0, 0.940]	0.82 [0.550, 0.930]	0.830 [0, 0.940]	<0.001
Missing	1 (0.1%)	1 (0.1%)	0 (0%)	0 (0%)	
**VOLSMTY**					
Median [Min, Max]	0.970 [0.760, 1.00]	0.980 [0.780, 1.00]	0.970 [0.760, 1.00]	0.960 [0.760, 1.00]	<0.001
Missing	1 (0.1%)	1 (0.1%)	0 (0%)	0 (0%)	
**HDRFDST**					
Median [Min, Max]	4.17 [1.37, 52.9]	3.98 [1.37, 52.9]	4.94 [1.92, 39.7]	4.74 [1.88, 37.0]	<0.001
Missing	1 (0.1%)	1 (0.1%)	0 (0%)	0 (0%)	
**AVGDIST**					
Median [Min, Max]	0.090 [0.030, 805]	0.080 [0.030, 805]	0.130 [0.03, 107]	0.120 [0.030, 15.1]	<0.001
Missing	1 (0.1%)	1 (0.1%)	0 (0%)	0 (0%)	
**LS**					
**DICE**					
Median [Min, Max]	0.920 [0.080, 0.970]	0.930 [0.260, 0.970]	0.90 [0.08, 0.960]	0.900 [0.260, 0.960]	<0.001
Missing	1 (0.1%)	1 (0.1%)	0 (0%)	0 (0%)	
**JACRD**					
Median [Min, Max]	0.860 [0.040, 0.950]	0.860 [0.150, 0.950]	0.830 [0.04, 0.920]	0.830 [0.150, 0.920]	<0.001
Missing	1 (0.1%)	1 (0.1%)	0 (0%)	0 (0%)	
**VOLSMTY**					
Median [Min, Max]	0.980 [0.120, 1.00]	0.980 [0.800, 1.00]	0.970 [0.120, 1.00]	0.960 [0.260, 1.00]	<0.001
Missing	1 (0.1%)	1 (0.1%)	0 (0%)	0 (0%)	
**HDRFDST**					
Median [Min, Max]	3.75 [1.33, 42.3]	3.75 [1.33, 41.3]	4.26 [1.88, 35.5]	4.42 [2.08, 42.3]	<0.001
Missing	1 (0.1%)	1 (0.1%)	0 (0%)	0 (0%)	
**AVGDIST**					
Median [Min, Max]	0.0900 [0.030, 9.54]	0.080 [0.030, 9.54]	0.110 [0.04, 4.80]	0.130 [0.0400, 1.70]	<0.001
Missing	1 (0.1%)	1 (0.1%)	0 (0%)	0 (0%)	

Table S8. Scanning protocols of the DWI/ADC images.

	Overall (N=2105)	train (N=1681)	validate (N=212)	test (N=212)	*P* value
**Magnetic Field**					
1.5T	316 (15.0%)	254 (15.1%)	36 (17.0%)	26 (12.3%)	0.384
3.0T	1789 (85.0%)	1427 (84.9%)	176 (83.0%)	186 (87.7%)	
**Manufacture**					
GE MEDICAL SYSTEMS	1206 (57.3%)	959 (57.0%)	127 (59.9%)	120 (56.6%)	0.409
Philips Medical Systems	226 (10.7%)	184 (10.9%)	15 (7.1%)	27 (12.7%)	
SIEMENS	571 (27.1%)	451 (26.8%)	62 (29.2%)	58 (27.4%)	
UIH	102 (4.8%)	87 (5.2%)	8 (3.8%)	7 (3.3%)	
**Model Name**					
Achieva	61 (2.9%)	52 (3.1%)	5 (2.4%)	4 (1.9%)	0.871
Aera	271 (12.9%)	217 (12.9%)	32 (15.1%)	22 (10.4%)	
Amira	2 (0.1%)	1 (0.1%)	1 (0.5%)	0 (0%)	
DISCOVERY MR750	1065 (50.6%)	844 (50.2%)	112 (52.8%)	109 (51.4%)	
DISCOVERY MR750w	95 (4.5%)	75 (4.5%)	13 (6.1%)	7 (3.3%)	
Ingenia	134 (6.4%)	107 (6.4%)	8 (3.8%)	19 (9.0%)	
Ingenia CX	1 (0.0%)	1 (0.1%)	0 (0%)	0 (0%)	
Multiva	30 (1.4%)	24 (1.4%)	2 (0.9%)	4 (1.9%)	
Prisma	35 (1.7%)	29 (1.7%)	2 (0.9%)	4 (1.9%)	
SIGNA EXCITE	42 (2.0%)	36 (2.1%)	2 (0.9%)	4 (1.9%)	
Signa HDxt	3 (0.1%)	3 (0.2%)	0 (0%)	0 (0%)	
SIGNA Premier	1 (0.0%)	1 (0.1%)	0 (0%)	0 (0%)	
Skyra	60 (2.9%)	46 (2.7%)	6 (2.8%)	8 (3.8%)	
TrioTim	149 (7.1%)	114 (6.8%)	17 (8.0%)	18 (8.5%)	
uMR 790	102 (4.8%)	87 (5.2%)	8 (3.8%)	7 (3.3%)	
Verio	54 (2.6%)	44 (2.6%)	4 (1.9%)	6 (2.8%)	
**B value (s/mm2)**					
Median [Q1, Q3]	1400 [1400, 1400]	1400 [1400, 1400]	1400 [1400, 1400]	1400 [1400, 1400]	0.703
**Repetition Time (ms)**					
Median [Q1, Q3]	2930 [2640, 4370]	3000 [2640, 4380]	2930 [2640, 4110]	2910 [2640, 4130]	0.762
**Echo Time (ms)**					
Median [Q1, Q3]	61.3 [59.7, 63.8]	61.3 [59.7, 63.8]	61.2 [60.0, 63.7]	61.3 [59.7, 63.5]	0.973
**Pixel Bandwidth (Hz)**					
Median [Q1, Q3]	1950 [1570, 1950]	1950 [1630, 1950]	1950 [1540, 1950]	1950 [1610, 1950]	0.212
**Reconstruction Diameter (mm)**					
Median [Q1, Q3]	240 [200, 240]	240 [200, 240]	240 [200, 240]	240 [220, 240]	0.344
**Slice Thickness (mm)**					
Median [Q1, Q3]	4.00 [4.00, 4.00]	4.00 [4.00, 4.00]	4.00 [4.00, 4.00]	4.00 [4.00, 4.00]	0.963
**Slice Spacing (mm)**					

Median [Q1, Q3]	4.00 [4.00, 4.50]	4.00 [4.00, 4.50]	4.00 [4.00, 4.50]	4.00 [4.00, 4.50]	0.869
**Pixel Spacing (mm)**					
Median [Q1, Q3]	0.938 [0.938, 1.30]	0.938 [0.938, 1.30]	0.938 [0.938, 1.30]	0.938 [0.938, 1.30]	0.809

Table S9. Segmentation metrics of the model (median [Q1, Q3]).

	Overall (N=2105)	train (N=1681)	validate (N=212)	test (N=212)	*P* value
**DSC**	0.920 [0.870, 0.940]	0.930 [0.910, 0.950]	0.810 [0.698, 0.870]	0.800 [0.615, 0.865]	<0.001
**JACRD**	0.850 [0.770, 0.890]	0.870 [0.830, 0.900]	0.675 [0.528, 0.770]	0.670 [0.445, 0.760]	<0.001
**VOLSMTY**	0.990 [0.950, 0.990]	0.990 [0.980, 1.00]	0.900 [0.800, 0.950]	0.870 [0.735, 0.950]	<0.001
**HDRFDST**	7.40 [3.08, 13.5]	5.63 [2.50, 12.4]	9.94 [6.76, 18.0]	12.5 [7.91, 20.4]	<0.001
**AD**	0.110 [0.0600, 0.300]	0.0800 [0.0500, 0.180]	0.400 [0.180, 1.11]	0.490 [0.225, 1.67]	<0.001

Table S10. Measurements of the manually labeled areas and the predicted areas (median [Q1, Q3]).

	Overall (N=2105)	train (N=1681)	validate (N=212)	test (N=212)	*P* Value
**Lesion number**	1.00 [0, 2.00]	1.00 [0, 2.00]	1.00 [0, 2.00]	1.00 [0, 3.00]	0.564
**pLesion number**	1.00 [1.00, 1.00]	1.00 [1.00, 1.00]	1.00 [1.00, 1.00]	1.00 [1.00, 1.00]	0.723
**Volume**	1.49 [0, 11.3]	1.53 [0, 11.1]	1.36 [0, 11.8]	1.60 [0, 12.7]	0.989
**pVolume**	3.12 [0.864, 13.1]	3.16 [0.864, 12.9]	2.99 [0.730, 14.3]	2.99 [1.06, 13.1]	0.953
**Intensity**	15300 [12900, 18100]	15400 [13000, 18100]	15200 [12800, 17800]	14800 [13100, 18100]	0.780
**pIntensity**	16100 [13400, 18800]	16100 [13400, 18900]	16200 [13200, 18600]	15600 [13200, 18400]	0.421
**DiaX**	2.12 [0, 4.10]	2.11 [0, 4.10]	1.88 [0, 4.12]	2.21 [0, 4.12]	0.986
**pDiaX**	2.69 [1.38, 4.20]	2.64 [1.37, 4.22]	2.82 [1.39, 4.11]	2.72 [1.62, 4.18]	0.916
**DiaY**	1.90 [0, 3.63]	1.91 [0, 3.56]	1.89 [0, 3.73]	1.84 [0, 3.94]	0.857
**pDiaY**	2.32 [1.34, 3.73]	2.31 [1.34, 3.67]	2.38 [1.25, 3.90]	2.32 [1.32, 3.85]	0.814
**DiaZ**	1.95 [0, 3.60]	1.95 [0, 3.60]	1.70 [0, 3.58]	1.94 [0, 3.97]	0.902
**pDiaZ**	2.41 [1.35, 3.98]	2.41 [1.32, 3.98]	2.44 [1.31, 3.77]	2.40 [1.61, 3.98]	0.702

Table S11. Bland–Altman analysis of the measured values of the prostate cancer.

	RL Diameter	AP Diameter	SI Diameter	Volume	Signal Intensity
bias	-0.464	-0.431	-0.526	-1.306	-0.464
biasUpperCI	-0.394	-0.37	-0.464	-1.053	-0.394
biasLowerCI	-0.533	-0.492	-0.588	-1.56	-0.533
biasStdDev	1.63	1.425	1.453	5.926	1.63
biasSEM	0.036	0.031	0.032	0.129	0.036
LOA_SEM	0.061	0.053	0.054	0.221	0.061
upperLOA	2.73	2.361	2.322	10.309	2.73
upperLOA_upperCI	2.85	2.465	2.428	10.742	2.85
upperLOA_lowerCI	2.611	2.257	2.216	9.876	2.611
lowerLOA	-3.657	-3.223	-3.374	-12.922	-3.657
lowerLOA_upperCI	-3.538	-3.119	-3.267	-12.489	-3.538
lowerLOA_lowerCI	-3.777	-3.327	-3.48	-13.355	-3.777
regression.fixed.slope	0.15	0.12	0.12	-0.02	0.15
regression.fixed.intercept	-0.83	-0.71	-0.82	-1.1	-0.83

LOA, limits of agreement; CI, confidence interval.

Figure S1. The modified Med3D network.

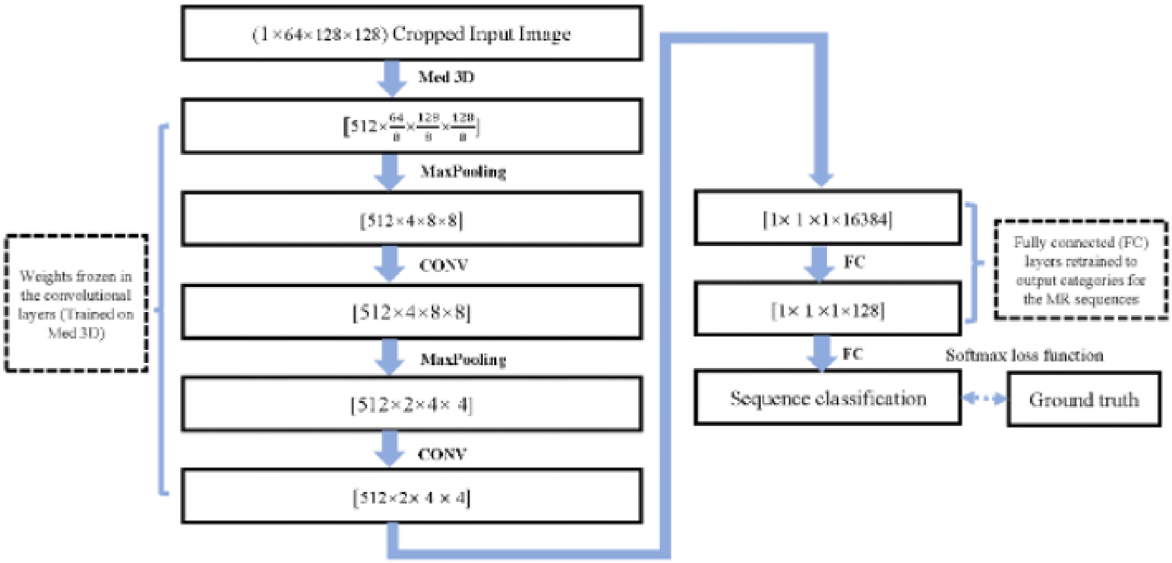

Figure S2. Confusion matrix of the prediction results in the training, validation, and testing datasets. The number in the middle of each tile is the counted number of images. The percentage number at the bottom of each tile is the column percentage. The percentage number at the right side of each tile is the row percentage. The color intensity is based on the counts.

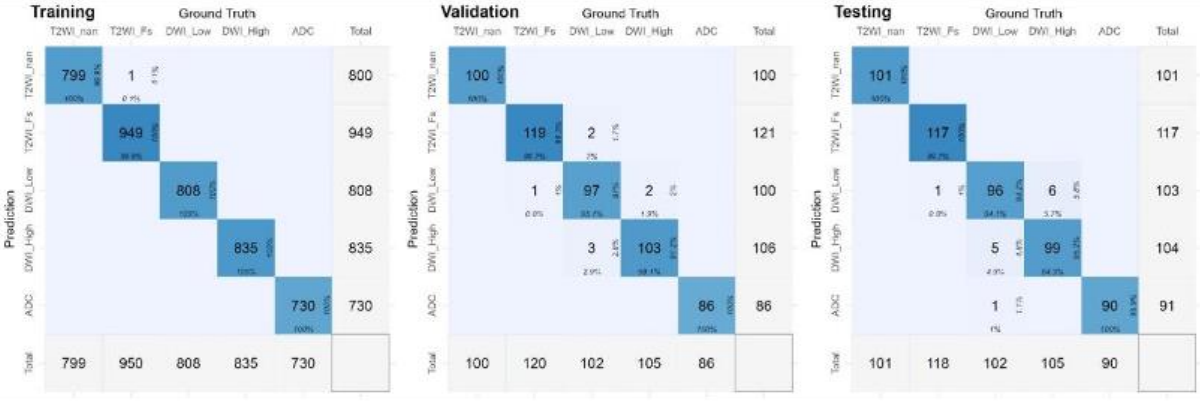

Figure S3. Whole prostate segmentation and the algorithm rule of the minimum volume bounding box.

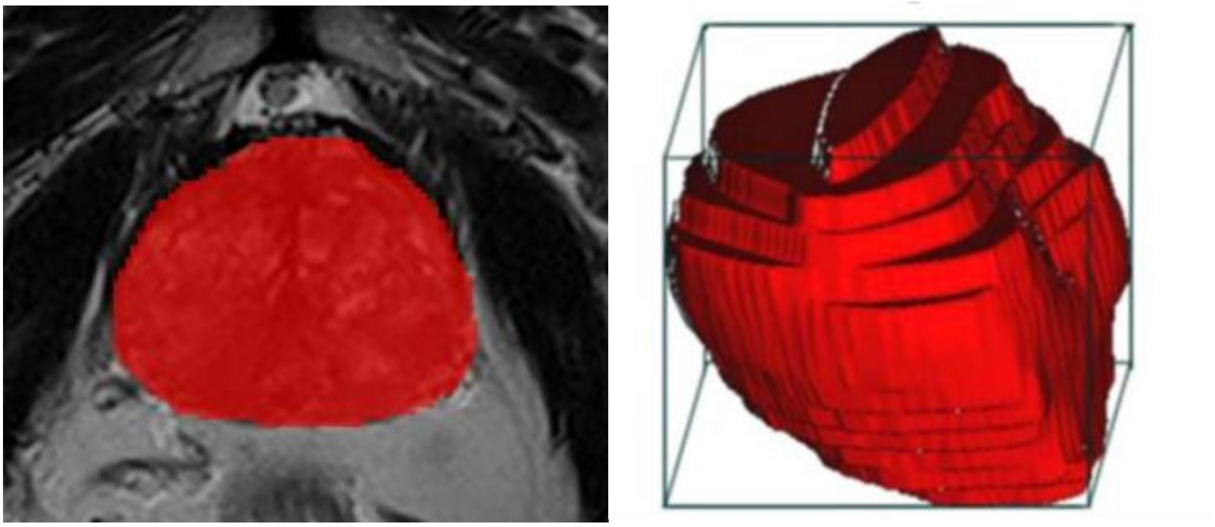

Figure S4. The DSC, Jacard index, VS, HD, and AD in different datasets. The metrics of the T2WI were superior to those of the ADC map in all datasets (all P<0.001). DSC Dice similarity coefficient, VS volumetric similarity, HD Hausdorff distance, and AD Average distance. T2WI T2-weighted imaging, ADC apparent diffusion coefficient.

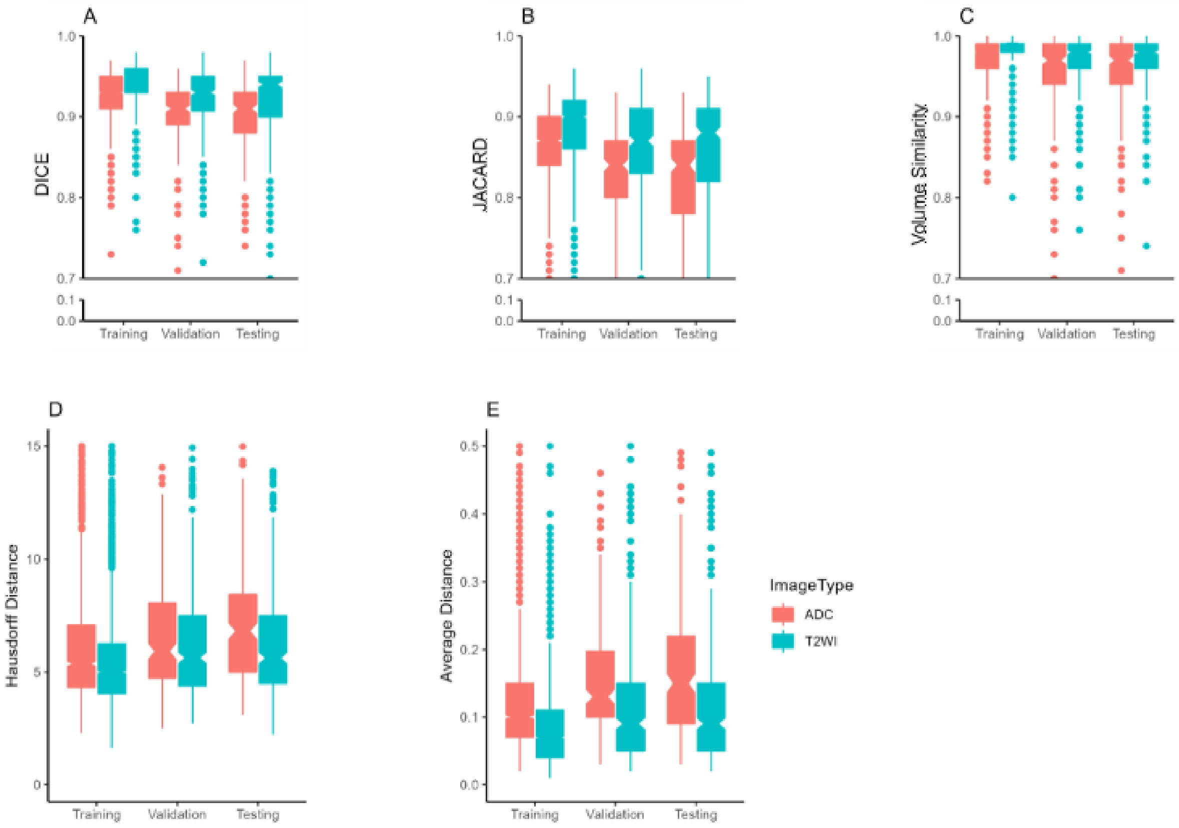

Figure S5. Bland–Altman analysis of the values of the RL diameter (A), AP diameter (B), SI diameter (C), volume (D), and signal intensity (E) of the manual label and the predicted label of the prostate gland. RL right and left, AP anterior and posterior, SI superior and inferior.

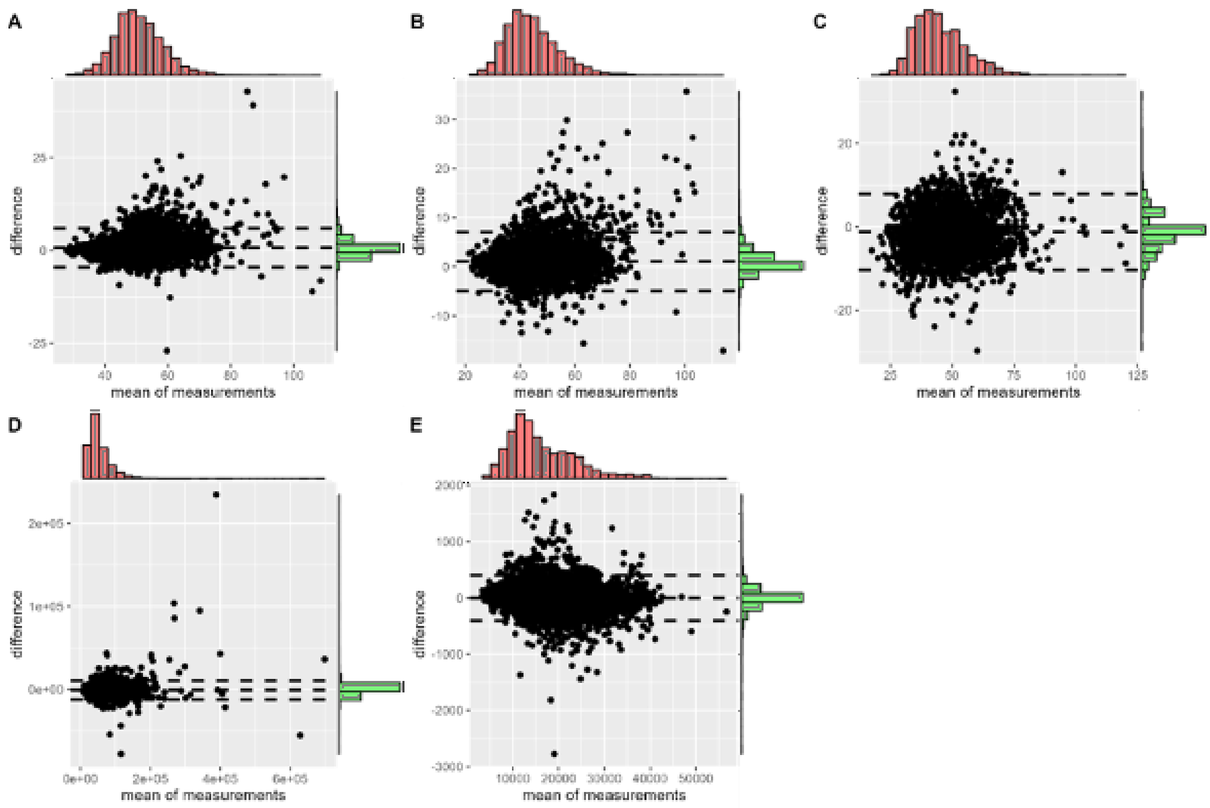

Figure S6. Sextant locations.

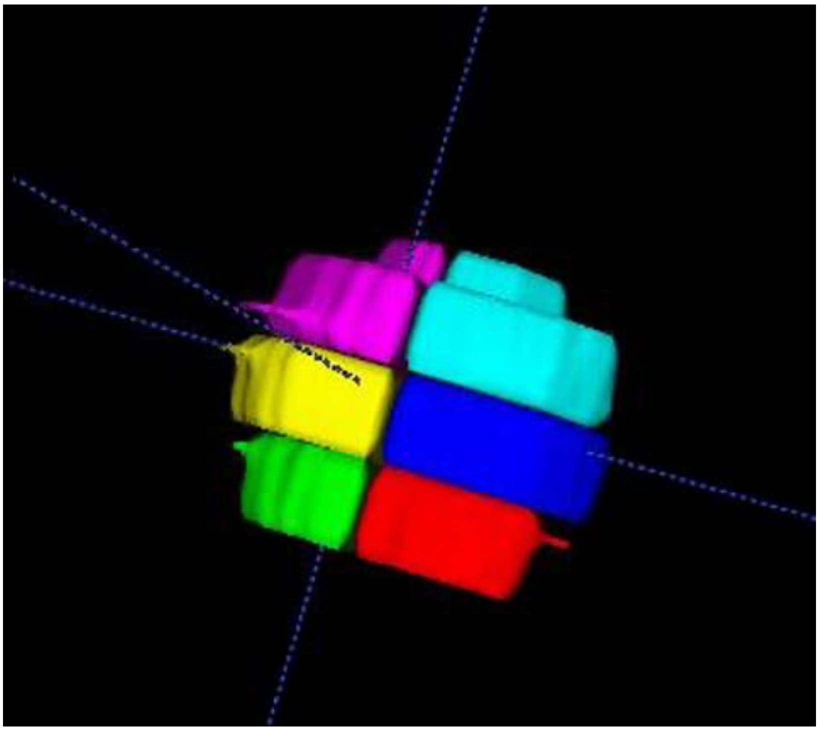

Figure S7. Anatomic zone locations.

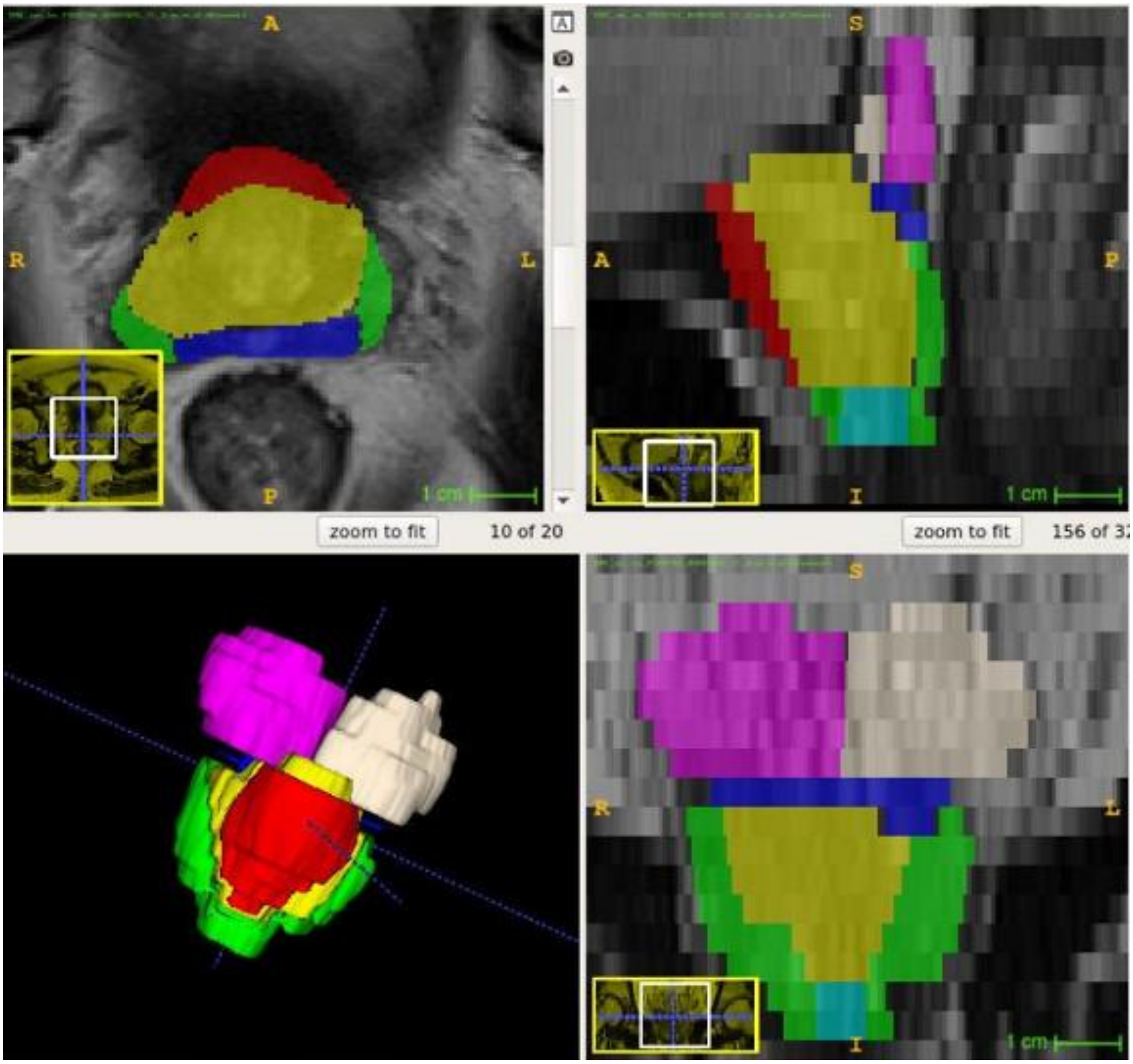

Figure S8. Segmentation metrics of the model, showing the DSC (A), Jacard index (B), VS (C), HD (D), and AD (E) of the training, validation, and test dataset.

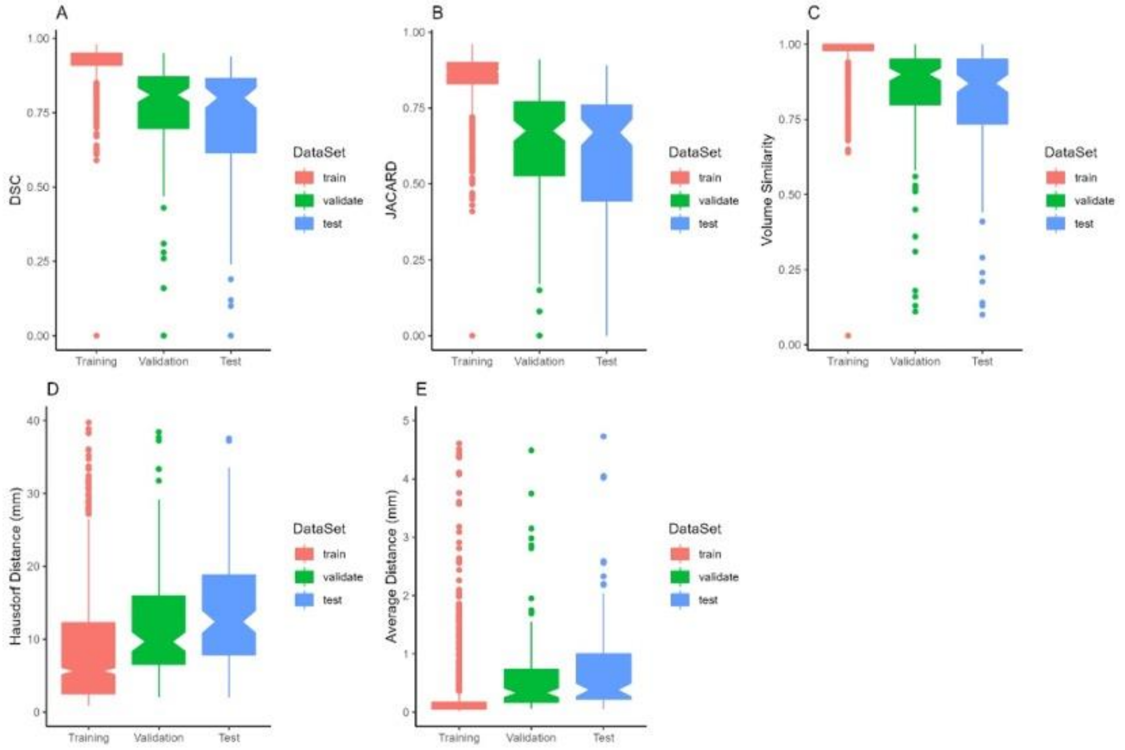

Figure S9. Bland–Altman analysis of the values of the RL diameter (A), AP diameter (B), SI diameter (C), volume (D), and signal intensity (E) of the manual label and the predicted label of the prostate cancer.

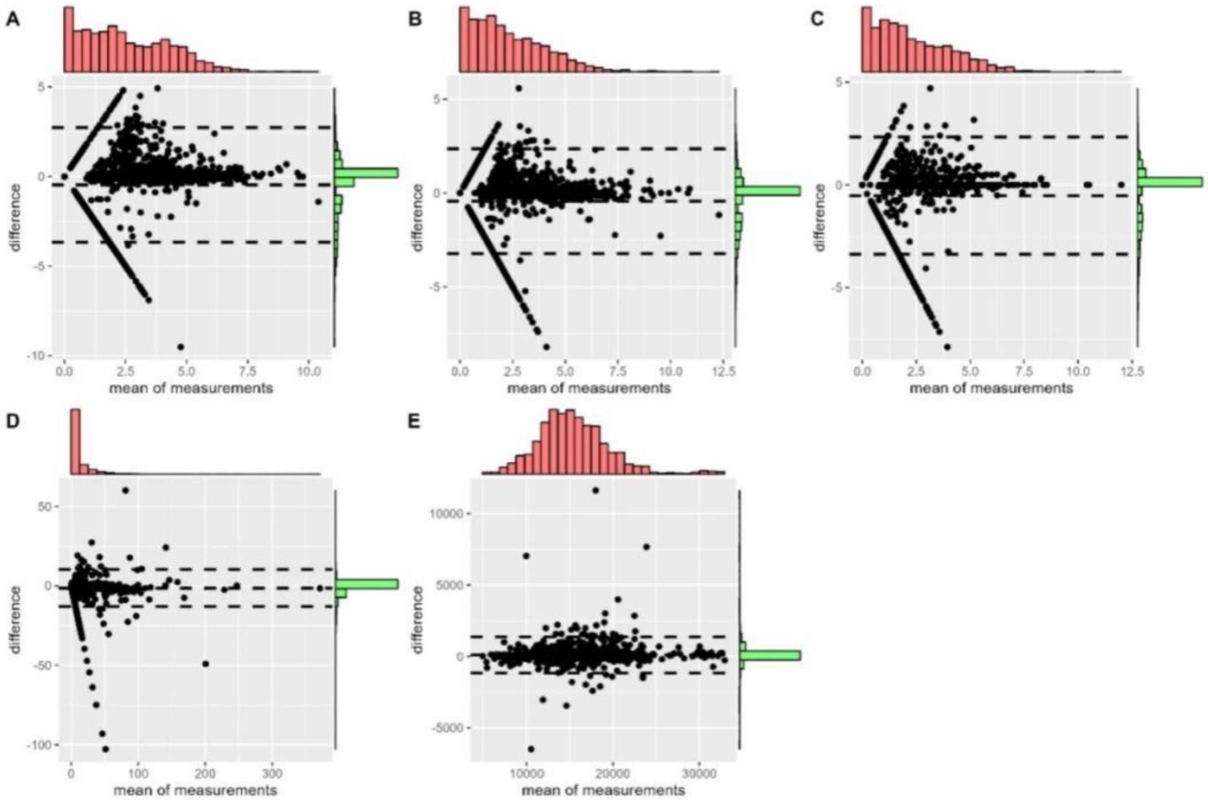

Supplement: Supplementary file 1 — Additional file 1: Information on the development and performance of the AI software. [file 13244_2023_1421_MOESM1_ESM.pdf]
